# Conservation implications of low contemporary connectivity along the Mid‐Atlantic Ridge in hydrothermal vent gastropods

**DOI:** 10.1111/cobi.70284

**Published:** 2026-04-24

**Authors:** E. Portanier, A. Tran Lu Y, F. Pradillon, C. Daguin‐Thiébaut, S. Ruault, P. Collins, E. Omnes, S. Fuchs, J. Carlsson, D. Jollivet, M. Matabos

**Affiliations:** ^1^ Sorbonne Université, CNRS, UMR 7144 ‘AD2M’, DISEEM group, Station Biologique de Roscoff Roscoff France; ^2^ Univ Brest, Ifremer, BEEP Plouzané France; ^3^ ISEM, Institut des Sciences de l'Evolution Université de Montpellier, CNRS, EPHE, IRD Montpellier France; ^4^ MARBEC UMR 248 Université de Montpellier, Ifremer, IRD, CNRS Sète France; ^5^ School of Biological Sciences Queen's University Belfast Belfast UK; ^6^ Area52 Research Group, School of Biology and Environmental Science/Earth Institute University College Dublin Dublin Ireland

**Keywords:** ddRAD, demographic inference, *Lepetodrilus atlanticus*, *Peltospira smaragdina*, population genomics, vent fauna conservation, Conservación de fauna de fuentes hidrotermales, ddRAD, genómica de poblaciones, inferencia demográfica *Peltospira smaragdina*, *Lepetodrilus atlanticus*

## Abstract

Polymetallic sulfide deposits produced at hydrothermal vent fields are targets for mining exploitation along the Mid‐Atlantic Ridge, threatening the functioning and resilience of vent ecosystems that provide multiple ecosystem services. Knowledge about connectivity between vents will inform conservation practices. The present‐day genetic structure and demographic history of the two vent‐dwelling gastropod species, *Lepetodrilus atlanticus* and *Peltospira smaragdina*, were investigated using more than 15,000 single‐nucleotide polymorphisms and mt*Cox1* sequences. Each species comprised three genetic groups. Genetic breaks were stronger for *L. atlanticus*, separating three distinct operational taxonomic units (5°S, Broken Spur at 29°N, and the Azorean vent fields near 37°N). *Peltospira smaragdina* was also geographically separated into three groups: Broken Spur, TAG, and Snake Pit (23–29°N); Lucky Strike (37°N); and Moytirra (45°N). A semipermeable zone was detected south of Rainbow (35°N), similar to that of vent mussels in this area, suggesting the presence of a multispecies hybrid zone. Demographic inferences supported secondary contact between most pairs of metapopulations for both species, but the time since contact was insufficient for allele frequencies to rehomogenize. Gene flow between vent fields may be sporadic and rare or restricted by genetic barriers. The fragmentation of species into isolated metapopulations may reduce their resilience to disturbance and create the need for specific conservation measures. Isolated populations (e.g., Moytirra for *P. smaragdina*, 5°S and Broken Spur for *L. atlanticus*), source populations (e.g., Lucky Strike for *P. smaragdina*), admixture areas, and sites that may act as stepping stones within a metapopulation must be protected from deep‐sea mining. An average distance of <100 km between favorable habitats in regional environmental management plans could help maintain the species’ genetic diversity and connectivity along the ridge.

## Abstract (es)

Implicaciones para la conservación de la escasa conectividad actual a lo largo de la cordillera meso atlántica en gasterópodos de las fuentes hidrotermales

**Resumen:**

Los yacimientos de sulfuros polimetálicos que se forman en los campos de fuentes hidrotermales son objeto de explotación minera a lo largo de la cordillera meso atlántica, lo que amenaza el funcionamiento y la resiliencia de los ecosistemas de las fuentes hidrotermales, que proporcionan múltiples servicios ecosistémicos. El conocimiento sobre la conectividad entre las fuentes hidrotermales servirá de base para las prácticas de conservación. Se investigó la estructura genética actual y la historia demográfica de las dos especies de gasterópodos que habitan en las fuentes hidrotermales, *Lepetodrilus atlanticus* y *Peltospira smaragdina*, utilizando más de 15,000 polimorfismos de un solo nucleótido y secuencias de mtCox1. Cada especie comprendía tres grupos genéticos. Las rupturas genéticas fueron más marcadas en *L. atlanticus*, separando tres unidades taxonómicas operativas distintas (5°S, Broken Spur a 29°N y los campos de fuentes hidrotermales de las Azores cerca de 37°N). P*eltospira smaragdina* también se separó geográficamente en tres grupos: Broken‐Spur, TAG, Snake Pit (23‐29°N), Lucky Strike (37°N) y Moytirra (45°N). Se detectó una zona semipermeable al sur de Rainbow (35° N), similar a la de los mejillones de las fuentes hidrotermales de esta zona, lo que sugiere la presencia de una zona híbrida multiespecífica. Las inferencias demográficas respaldaron el contacto secundario entre la mayoría de los pares de metapoblaciones de ambas especies, pero el tiempo transcurrido desde el contacto fue insuficiente para que las frecuencias alélicas se rehomogeneizaran. El flujo genético entre los campos de fuentes hidrotermales puede ser esporádico y poco frecuente o estar restringido por barreras genéticas. La fragmentación de las especies en metapoblaciones aisladas puede reducir su resiliencia ante las perturbaciones y crear la necesidad de medidas de conservación específicas. Las poblaciones aisladas (por ejemplo, Moytirra para *P. smaragdina*, 5°S y Broken‐Spur para *L. atlanticus*), las poblaciones de origen (por ejemplo, Lucky Strike para *P. smaragdina*), las zonas de mestizaje y los sitios que puedan actuar como puentes dentro de una metapoblación deben protegerse de la minería en aguas profundas. Una distancia media inferior a 100 km entre hábitats favorables en los planes regionales de gestión medioambiental podría ayudar a mantener la diversidad genética y la conectividad de las especies a lo largo de la cordillera.

## INTRODUCTION

Hydrothermal vents represent oases of life hosting high biomass of endemic species dependent on chemosynthesis (Gebruk et al., 2000; Van Dover et al., 2018). In these areas, the metal‐rich hydrothermal fluids lead to the formation of polymetallic sulfides, which are of increasing interest for the mining industry (Petersen et al., 2016). The impacts of anthropogenic activities on hydrothermal ecosystems are expected to be substantial and include biodiversity and habitat loss, changes in the shape and texture of the seafloor, alteration of the hydrothermal fluid circulation, creation of mineral particle plumes at the bottom and mid‐water regions, and noise and light pollution (Amon et al., 2022; Gollner et al., 2017; Ramirez‐Llodra et al., 2011; Van Dover, 2014). Such disturbances can have irreversible effects on deep ocean ecosystem services (e.g., climate regulation, nutrient cycling, resources, Levin et al., 2020) that would add to those expected from other anthropogenic constraints (climate change, pollution, harvesting) (Gollner et al., 2021; Van Dover et al., 2018). Along the Mid‐Atlantic Ridge (MAR), mining exploration authorizations have been granted by the International Seabed Authority (ISA) (Allcock et al., 2025; Blanchard & Gollner, 2022). It is thus urgent to better characterize the functioning and resilience of hydrothermal communities to properly inform conservation measures.

Genetic and demographic connectivity (i.e., the degree to which gene flow affects evolutionary processes within populations and the degree to which population growth and vital rates are affected by dispersal, respectively [Lowe & Allendorf, 2010]) are of paramount importance for ecosystem resilience (Baco et al., 2016; Gollner et al., 2017; Lynch et al., 1995; Mullineaux et al., 2018) because they contribute to the distribution of species and the maintenance of genetic variation and adaptive potential. Deep‐sea mining may directly disrupt dispersal by increasing adult or larval mortality, altering reproduction, and reducing fecundity, which can affect recruitment success (Carreiro‐Silva et al., 2022). It may also lead to the loss of stepping‐stone sites that play a crucial role in maintaining connectivity. Characterizing recent and historical genetic connectivity (i.e., demographic history) of species inhabiting hydrothermal vents is, therefore, essential for science‐based deep‐sea management.

In the open ocean, the impacts of hydrodynamics on connectivity have been well studied (e.g., Hellberg, 2009; Jahnke & Jonsson, 2022; White et al., 2010). For benthic species, long‐distance dispersal often occurs via passive transport of larvae or brooding adults (e.g., vent copepods or amphipods) by ocean currents (Cowen & Sponaugle, 2009; Dibacco et al., 2006). Because direct monitoring of propagules is difficult (minute size, dilution in large water volumes; Cowen & Sponaugle, 2009), indirect measures of connectivity derived from population genetics approaches are useful for the design of conservation plans. These approaches inform about patterns of gene flow and pivotal sites for the maintenance of faunal communities (e.g., source populations that replenish other sites, sites that show unique gene pools, or sites that serve as stepping stones along dispersal routes; Baco et al., 2016; Balbar & Metaxas, 2019; Palumbi, 2003; Pujolar et al., 2013; Taboada et al., 2018).

Most studies assessing population genetic connectivity of hydrothermal vent fauna used a limited number of genetic markers (e.g., Plouviez et al., 2008; Teixeira et al., 2013; Thaler et al., 2011), although, recently, next‐generation sequencing approaches have been applied (e.g., Bracco et al., 2019; Diaz‐Recio Lorenzo et al., 2024; Plouviez et al., 2019; Tran Lu Y et al., 2022, 2025). Many of them focused on a single species, whereas geography, ridge topology, or habitat fragmentation may differently affect the population genetic structure and colonization history of different species, depending on their life‐history traits (e.g., louviez et al., 2009; Poitrimol et al., 2022; Thaler et al., 2014; Tran Lu Y, 2022). Reproductive strategy, fecundity, as well as the larval swimming ability, nutritional requirements, and position in the water column may differ across species and determine which features constitute barriers to dispersal. Therefore, environmental management requires multispecies comparative approaches (Baco et al., 2016; Magris et al., 2018; Nielsen et al., 2017; Paz‐Vinas et al., 2018). Such studies, conducted along the East Pacific Rise (EPR) and the western Pacific back‐arc basins (Plouviez et al., 2009; Tran Lu Y et al., 2025), highlighted the role of plate tectonics as a driver of vicariance events leading to the actual biogeographic patterns (Matabos et al., 2011; Matabos & Jollivet, 2019).

Along the MAR, most genetic studies focused on *Bathymodiolus* vent mussels (Breusing et al., 2016; O'Mullan et al., 2001; van der Heijden et al., 2012) and *Rimicaris* shrimp (Creasey et al., 1996; Teixeira et al., 2011, 2013), and few genetic markers were used. Along the Northern MAR, the genetic connectivity of gastropods has only been investigated for *Divia briandi* limpets (Yahagi et al., 2019). The peltospirid *Peltospira smaragdina* and the lepetodrilid *Lepetodrilus atlanticus* nevertheless form dense assemblages emblematic in this area. Due to its occurrence only in the northern Atlantic and its highly fragmented population, *P. smaragdina* is, in addition, listed as near threatened on the Vent Red List for mollusks (Thomas et al., 2021) and requires specific attention. Investigating the genetic connectivity of these species would contribute to a better understanding of vent connectivity at a large geographic scale.

In both species, a continuous reproduction and planktonic dispersal strategy may be expected, suggesting that *L. atlanticus* and *P. smaragdina* may be early colonizers after disturbances, playing an important role in the functioning and resilience of vent ecosystems (Bayer et al., 2011; Cuvelier et al., 2025; Marticorena et al., 2021; Matabos & Thiebaut, 2010; Mullineaux et al., 2010; Sarrazin et al., 2022). The massive recruitment event observed for *P. smaragdina* at the recently reactivated Broken Spur vent field in 2018 (D. Jollivet, personal observation) also supports this hypothesis. Larvae of *P. smaragdina* and its close relative *Peltospira gargantua* (Chen et al., 2025) have been collected near vents, close to the seafloor (F. Pradillon, personal observation). Although yet unknown in *P. smaragdina*, fecundity is relatively low in *L. atlanticus*, and lecithotrophic larval development may be expected for hydrothermal gastropods (Matabos & Thiebaut, 2010; Tyler et al., 2008). *Peltospira smaragdina* colonizes mineral and microbial mats that develop on bare substrata in areas of shimmering fluid and can form assemblages that reach up to 68,000 individuals/m^2^ (Sarrazin et al., 2022). *Lepetodrilus atlanticus* feeds on free‐living filamentous microorganisms (De Busserolles et al., 2009) and mostly inhabits *Bathymodiolus* mussel shells (a rare habitat compared to areas of shimmering fluid), except at the Menez Gwen vent field, where *P. smaragdina* is absent and where *L. atlanticus* also forms dense assemblages (i.e., up to 69,000 individuals/m^2^) on friable substratum covered by microbial mats.

This study aimed to identify the population structures of *P. smaragdina* and *L. atlanticus*, determine the extent to which populations are connected and when they separated, and reveal the demographic regions that warrant prioritized conservation attention along the MAR, considering the emerging threats of deep‐sea mining. To address these aims, we used the *Cox1* mitochondrial gene (mt*Cox1*) and double‐digest Restriction site Associated DNA sequencing (ddRAD‐seq) on individuals collected between 5°S and 38°N for *L. atlanticus* and between 23°N and 45°N for *P. smaragdina*, covering the entire known range of both species (Sarrazin et al., 2022). This allowed testing for the presence of taxonomic operational units, performing population genetic analyses, and making demographic inferences based on thousands of single‐nucleotide polymorphic sites (SNPs).

## METHODS

### Sample collection and DNA extraction

**FIGURE 1:**
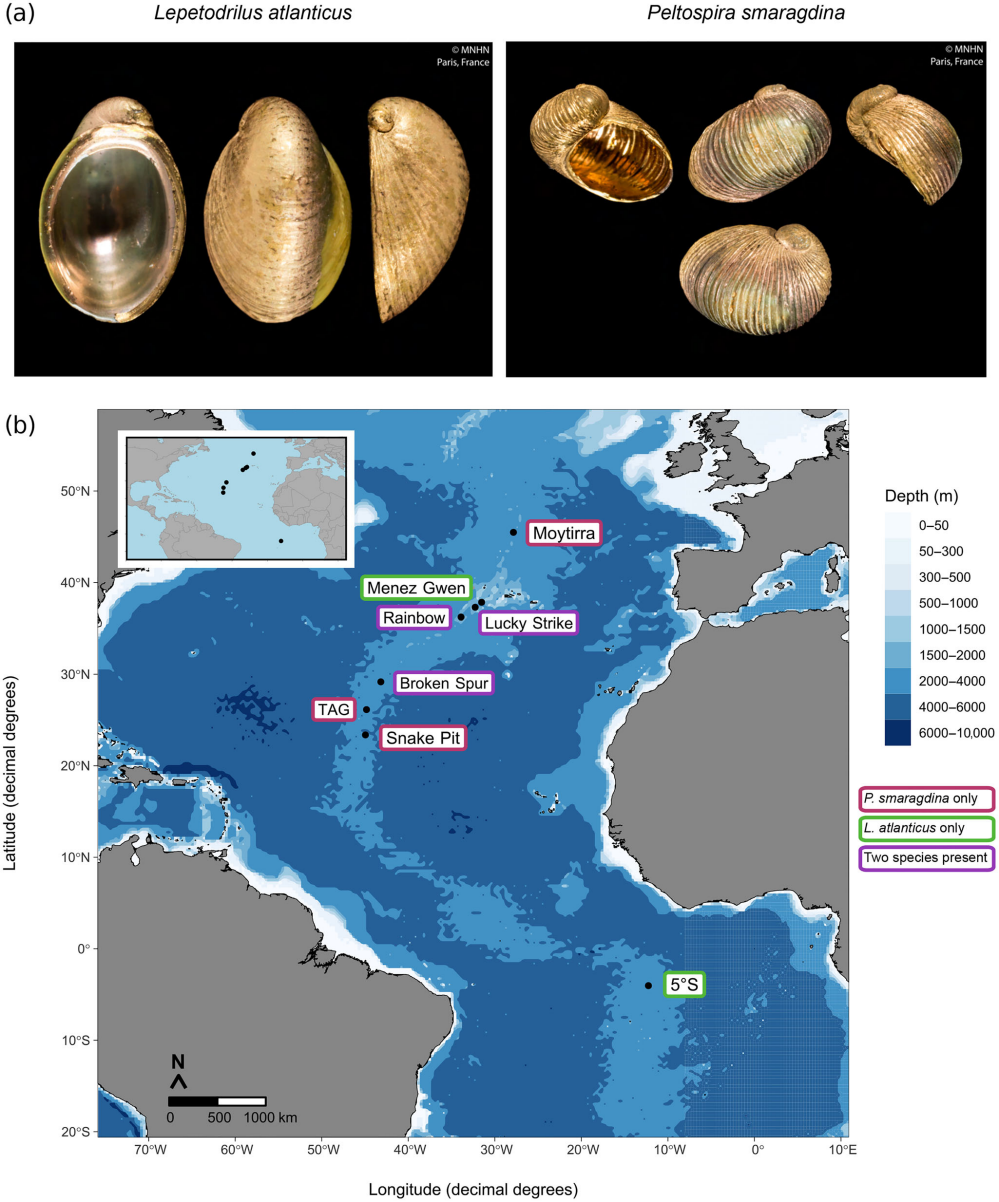
(a) *Lepetodrilus atlanticus* and *Peltospira smaragdina* in situ (photos by IFREMER) (top) and holotype specimens (photos by Manuel Caballer, 2016, MNHN, CC‐BY‐ 4.0) (bottom) and (b) locations of sampled hydrothermal vent fields along the north and south Mid‐Atlantic Ridge. Spatial coordinates of hydrothermal vent fields are in Appendix S1.

The sampling of 115 *L. atlanticus* from five hydrothermal vent sites (3000 to 840 m of depth) and 207 *P. smaragdina* from six sites (3600 to 1680 m of depth) occurred during eight scientific cruises with the human‐occupied vehicle *Nautile* or the remotely operated vehicles *Victor6000*, *Holland I*, and *Kiel 6000*, on board the research vessels *L'Atalante*, *Le Pourquoi‐Pas*, *James Cook*, and *Meteor* (Figure 1; Appendices S1–S3). Specimens were preserved in 80% molecular‐grade EtOH or frozen at −80°C after their recovery on board. DNA was extracted from entire animals after shell removal with a 2% CTAB (cetyltrimethyl ammonium bromide)–1% PVP (poly‐*N*‐vinyl‐2‐pyrrolidone) buffer following Jolly et al. (2003). DNA pellets were dried using a SpeedVac (ThermoFisher Scientific) and resuspended in 30–70 µL (according to the size of the pellet) of 0.1X Tris‐EDTA buffer (TE, pH 8.0). The quality of DNA samples was checked using electrophoresis on a 0.8% agarose gel stained with ethidium bromide and quantified by the Nanodrop ND‐1000 spectrophotometer (Nanodrop Technologies).

### Sequencing of mtDNA Cox1

The mt*Cox1* gene was amplified using the universally applicable Folmer primers LCO1490 and HCO2198 (Folmer et al., 1994) on a subsample of individuals (Appendix S4). Polymerase chain reactions (see details in Appendix S5) were performed, and both DNA strands were Sanger‐sequenced at the Macrogen laboratory. Individual chromatograms were checked, edited when necessary (e.g., trimmed), and assembled into a consensus sequence with Geneious 9.1.8. Consensus sequences were aligned and trimmed within each species with Seaview 4.7 (Edgar, 2004; Gouy et al., 2010). Exploitable sequences were obtained for 34 *L. atlanticus* and 73 *P. smaragdina* individuals from all vent sites (Appendix S4). Two additional sequences of *P. smaragdina* from GenBank were added to this dataset: Rainbow (accession GQ160764.1) and Lucky Strike (accession MH837538.1) (Lee et al., 2019).

### Library preparation and sequencing of ddRAD‐seq

Individual ddRAD libraries were prepared according to Brelsford et al. (2016) as modified by Daguin‐Thiébaut et al. (2021). For each individual, approximately 100 ng of DNA was digested using *PstI* and *MseI* high‐fidelity restriction enzymes (New‐England BioLabs). DNA fragments were then ligated, purified, amplified, and pooled together (see Daguin‐Thiébaut et al. [2021] for details). Fragments between 300 and 800 bp were selected within a 1.5% dye‐free marker *K* agarose gel cassette in a Pippin Prep run (Sagescience). The quality of each library was checked using a Bioanalyzer High Sensitivity DNA Chip (Agilent), following the manufacturer's instructions. Pooled libraries were paired‐end sequenced (150PE) by Novogene or Genoscope (Centre National de Séquençage) with NovaSeq 6000 or HiSeq 4000 Illumina sequencers. To allow genotyping error rate estimation, seven *L. atlanticus* and 11 *P. smaragdina* individuals that had high‐molecular‐weight DNA (improving the probability of obtaining good‐quality sequencing) were independently sequenced three times. Each plate comprised several negative controls to ensure the absence of contamination by foreign DNA.

### De novo assembly and SNP calling and filtering

Reads were checked for quality with FastQC 0.11.9 (Andrews, 2010) and demultiplexed using process_radtags in Stacks 2.52 (Catchen et al., 2011; Rochette et al., 2019) to remove adapter sequences (two mismatches allowed). The *‐c*, *‐q*, and *‐r* options were implemented to, first, remove any reads with an uncalled base; second, discard reads with low‐quality scores; and third, retrieve barcodes and RAD tags. A de novo assembly was performed, due to the lack of a reference genome, for each species. The *M* and *n* parameters were set to 5 and 3 for *L. atlanticus* and *P. smaragdina*, respectively (Appendix S6), after implementation of the *r0.80* optimization procedure on a subset of individuals, as detailed in Paris et al. (2017) and Rochette and Catchen (2017) (see Appendix S6 for details). Modules of the *Stacks* pipeline were then run sequentially on the dataset including all sequenced individuals, excluding cstacks, because we used the loci catalog obtained from the samples used for parameter optimization (i.e., sstacks with catalog option). The population module was run with *‐r* = 0.80 for both species, achieving a satisfying compromise between the number of SNPs and missing data for *L. atlanticus*. For *P. smaragdina*, we also applied *‐p* = 6 to keep only SNPs that were shared by all the sampled vent fields, as missing data were otherwise high for Lucky Strike individuals, probably due to population divergence, because the quality and number of reads were in the same order of magnitude for these individuals and others. For both species, the maximum observed heterozygosity and the minimum alternative allele count required to keep a SNP at a locus were set to 0.75 and 10, respectively, to avoid paralog assembly.

We used VCFtools 0.1.16 (Danecek et al., 2011) and R 4.1.0 (R Core Team, 2021) to eliminate potential unfiltered paralogs, SNPs, and individuals with more than 10% of missing data, as well as SNPs with a coverage lower than 10 times and those erroneously genotyped between triplicated samples (details in Appendix S6). Short‐distance linkage disequilibrium between SNPs was avoided by keeping only the first SNP of each locus. Finally, outlier SNPs were eliminated using the R package pcadapt 4.3.3 (Luu et al., 2017; Privé et al., 2020), with four principal components for each species (details in Appendices S7 & S8). We removed all SNPs with significant *p* values after *p* values were adjusted using either *q* values, Benjamini and Hochberg (1995) corrections, or Bonferroni corrections. Filtered VCFs were produced using VCFtools individuals and SNPs whitelists.

### Population genetic analyses on mitochondrial data

The assemble‐species‐by‐automatic‐partitioning method (ASAP) (Puillandre et al., 2021) implemented on the web (https://bioinfo.mnhn.fr/abi/public/asap/asapold.html) was applied on mt*Cox1* sequences to determine if several operational taxonomic units (OTUs) were present in each species. Default parameters and pairwise sequence distances calculated under the K2P substitution model were used. Relationships between haplotypes determined using DnaSP 6 (Rozas et al., 2017) were visualized with a minimum spanning haplotype network (Bandelt et al., 1999) constructed for each species with PopArt 1.7 (Leigh & Bryant, 2015). We used DnaSP 6 to infer haplotype (Hd) and nucleotide (π) diversities, the number of variable sites (*S*), the total number of mutations (Eta), and the average number *k* of nucleotide differences between haplotypes (Tajima, 1983). The net (Da) and total (Dxy) population divergences (Nei, 1987) were calculated between vent localities.

### Population genetic analyses on nuclear data

Using SNP datasets, principal component analyses (PCAs) were performed (*R* package *adegenet* 2.1.3; Jombart, 2008; Jombart & Ahmed, 2011). Pairwise fixation indices (*F*_ST_) (Pembleton et al., 2013; Weir & Cockerham, 1984) were calculated with StAMPP R 1.6.3 and tested against zero with loci resampling (*n* = 1000 bootstraps) to obtain confidence intervals. Observed and expected heterozygosity, nucleotide diversity (π), and heterozygote deviations from Hardy–Weinberg proportions (as estimated by *F*_IS_) were calculated with the populations module of Stacks with all sites from all ddRAD tags of the final dataset. The maximum‐likelihood approach implemented in ADMIXTURE 1.3 (Alexander & Lange, 2011; Zhou et al., 2011) was used to perform coancestry analyses. Datasets were formatted using PLINK 1.9 (Chang et al., 2015; Purcell et al., 2007). ADMIXTURE was run 10 times, with default parameters, for *K* values from 1 to 10, and 10‐fold cross‐validation (CV) was then applied to identify the *K* value minimizing the average CV error value. For this optimal *K* value, independent runs were combined using CLUMPP (Jakobsson & Rosenberg, 2007) as implemented in the online CLUMPAK server (https://clumpak.tau.ac.il/; Kopelman et al., 2015).

### Demographic history on nuclear data

We used a modified version of dadi 2.1 (Gutenkunst et al., 2009; see DATA AVAILABILITY STATEMENT) to infer past and present gene flow between pairs of populations. Dadi simulates joint allele frequency spectra (JAFS) according to different demographic scenarios and uses these JAFS for demogenetic inferences. Following Rougeux et al. (2017), Momigliano et al. (2021), and Tran Lu Y et al. (2022), we considered 28 demographic scenarios derived from four basic models: strict isolation (SI), isolation with migration (IM), ancient migration (AM), or secondary contact (SC) with a folded JAFS (see DATA AVAILABILITY STATEMENT for scripts). Each model represented a situation in which an ancestral population of size *N*_anc_ splits into two sister populations of effective size *N*_1_ and *N*_2_, respectively. Depending on the population model selected, the software infers the time of initial population splitting, as well as other times to indicate whether gene flow occurred in the past and stopped or was recently renewed following secondary contact. It can provide additional parameters such as the population growth rate and heterogeneities in effective population size and migration rate along the genome (Table 3; Appendix S9). Each of the 28 models was run 30 times independently to check for congruence (i.e., similar parameter estimates at least for 10 replicates). The best‐supported models were selected using the Akaike information criterion (AIC), and for these models, the estimated demographic parameters were converted to biological units (details of calculations in Appendix S10) with a mutation rate per site per generation (μ = 10^−8^) (Lynch, 2010; Tran Lu Y et al., 2022). Standard deviations of parameter estimates were calculated using the Fisher information matrix method implemented in dadi.

To estimate divergence times accurately, pairs of populations showing low proportions of shared alleles (i.e., populations genetically differentiated) should be considered. In addition, the presence of genetic substructure within a group of individuals considered in dadi can bias immigration rate estimation (Le Moan et al., 2016; Rougemont et al., 2017; Rougeux et al., 2017). A unique hydrothermal vent field from each genetic cluster was thus included. Menez Gwen (*L. atlanticus*) and Snake Pit (*P. smaragdina*) were chosen to represent their respective clusters (see RESULTS). These populations were the most geographically distant from possible contact zones where the juxtaposition of parental types and hybrids would prevent a good fit of models. Because dadi is based on the assumption that loci are not in linkage disequilibrium, Rainbow (possible hybrid zone) was excluded from demographic inferences for *P. smaragdina* (Le Moan et al., 2016). Observed JAFS were thus built for three pairs of populations for each species: Broken Spur–5°S, Broken Spur–Menez Gwen, and 5°S–Menez Gwen for *L. atlanticus* (Appendix S11) and Moytirra–Lucky Strike, Moytirra–Snake Pit, and Lucky Strike–Snake Pit for *P. smaragdina* (Appendix S11). We did not use the three‐population analysis developed in dadi because it does not account for linked selection and heterogeneity in the migration rate along the genome to detect barrier loci (overparameterization of the population model) and because it was highly dependent on the chronology of population splitting.

## RESULTS

### Cryptic species delimitation with mtDNA barcoding

**FIGURE 2:**
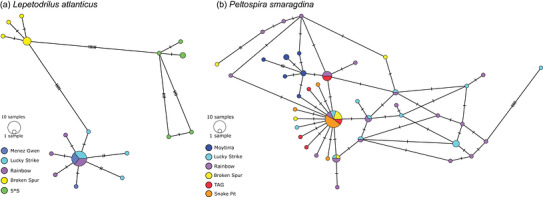
The mt*Cox1* haplotype networks for (a) *Lepetodrilus atlanticus* and (b) *Peltospira smaragdina* (circles, different haplotypes identified; circle size, proportional to number of individuals carrying the specific haplotype; color portions of circles, proportion of individuals carrying each haplotype in the different vent fields sampled; sequences were 657‐bp long; ticks on the branches, number of nucleotide substitutions between nucleotide sequences).

After alignment, mt*Cox1* sequences of 657 bp for both species revealed 17 and 41 haplotypes for *L. atlanticus* and *P. smaragdina*, respectively. Haplotype and nucleotide diversity were similar (Appendix S12). The haplotype network for *L. atlanticus* showed three geographic lineages separated by five to 10 nucleotide substitutions between sequences, corresponding to 5°S MAR, Broken Spur (29°N MAR), and the Azorean vent fields (Menez Gwen, Lucky Strike, and Rainbow) (Figure 2a). The 5°S MAR population appeared genetically structured with two divergent haplotypes (four differences between nucleotide sequences each) (Figure 2a). These groups were corroborated by ASAP analyses, which identified the presence of three distinct OTUs based on the distribution of pairwise differences (Appendix S13). In the most likely partition, the first group included all 5°S individuals, the second all individuals from Broken Spur, and the third all individuals from the Azorean populations. The level of divergence (Dxy) between 5°S MAR, Broken Spur, and the Azorean populations was between 1% and 2%, and lower between the Azorean populations (Table 1).

**TABLE 1:** Net population divergence (Da) (above diagonal) and total population divergence (Dxy) (below diagonal) for *Lepetodrilus atlanticus* and *Peltospira smaragdina* populations calculated from mt*Cox1* gene.

*Lepetodrilus atlanticus*						
		Da				
		Menez Gwen	Lucky Strike	Rainbow	Broken Spur	5°S
Dxy	Menez Gwen		0.0000	0.0000	0.0088	0.0134
	Lucky Strike	0.0013		0.0000	0.0088	0.0133
	Rainbow	0.0009	0.0015		0.0091	0.0136
	Broken Spur	0.0098	0.0104	0.0104		0.0096
	5°S	0.0161	0.0166	0.0165	0.0126	

*Peltospira smaragdina*							
		Da					
		Moytirra	Lucky Strike	Rainbow	Broken Spur	TAG	Snake Pit
Dxy	Moytirra		0.0032	0.0022	0.0023	0.0018	0.0027
	Lucky Strike	0.0069		0.0002	0.0007	0.0012	0.0013
	Rainbow	0.0058	0.0046		0.0003	0.0007	0.0011
	Broken Spur	0.0054	0.0046	0.0040		0.0001	0.0001
	TAG	0.0043	0.0045	0.0039	0.0027		0.0002
	Snake Pit	0.0046	0.0040	0.0037	0.0021	0.0015	

For *P. smaragdina*, the geographic structure was less pronounced (Figure 2b), as also illustrated by a lower average number of nucleotide differences (Appendix S13). Moytirra individuals showed private haplotypes, whereas other populations shared the most frequent one. Several haplotypes were only shared between the Rainbow and Lucky Strike populations. Moytirra haplotypes only slightly differed from those found in the other MAR populations (i.e., one to four differences between nucleotide sequences). The lowest ASAP score was for a two‐group partition, but the unimodal pairwise difference distribution (no gap) supported the presence of a unique OTU with only one individual from Lucky Strike assigned to the second group (Appendix S14). The Dxy between populations remained <1% with higher values when including the Moytirra population (Table 1).

### Population genetic structure with ddRAD‐seq data

The final datasets included 15,529 SNPs (mean coverage 29.93X) for *L. atlanticus* and 16,603 SNPs (mean coverage 24.86X) for *P. smaragdina*. Genetic diversity indices were almost similar between populations within species and between species. Exceptions regarded the 5°S MAR (*L. atlanticus*) and Moytirra (*P. smaragdina*) populations, which showed the highest (*H*_o_ = 0.244) and lowest heterozygosity (*H*_o_ = 0.140) values, respectively (Appendix S15).

**FIGURE 3:**
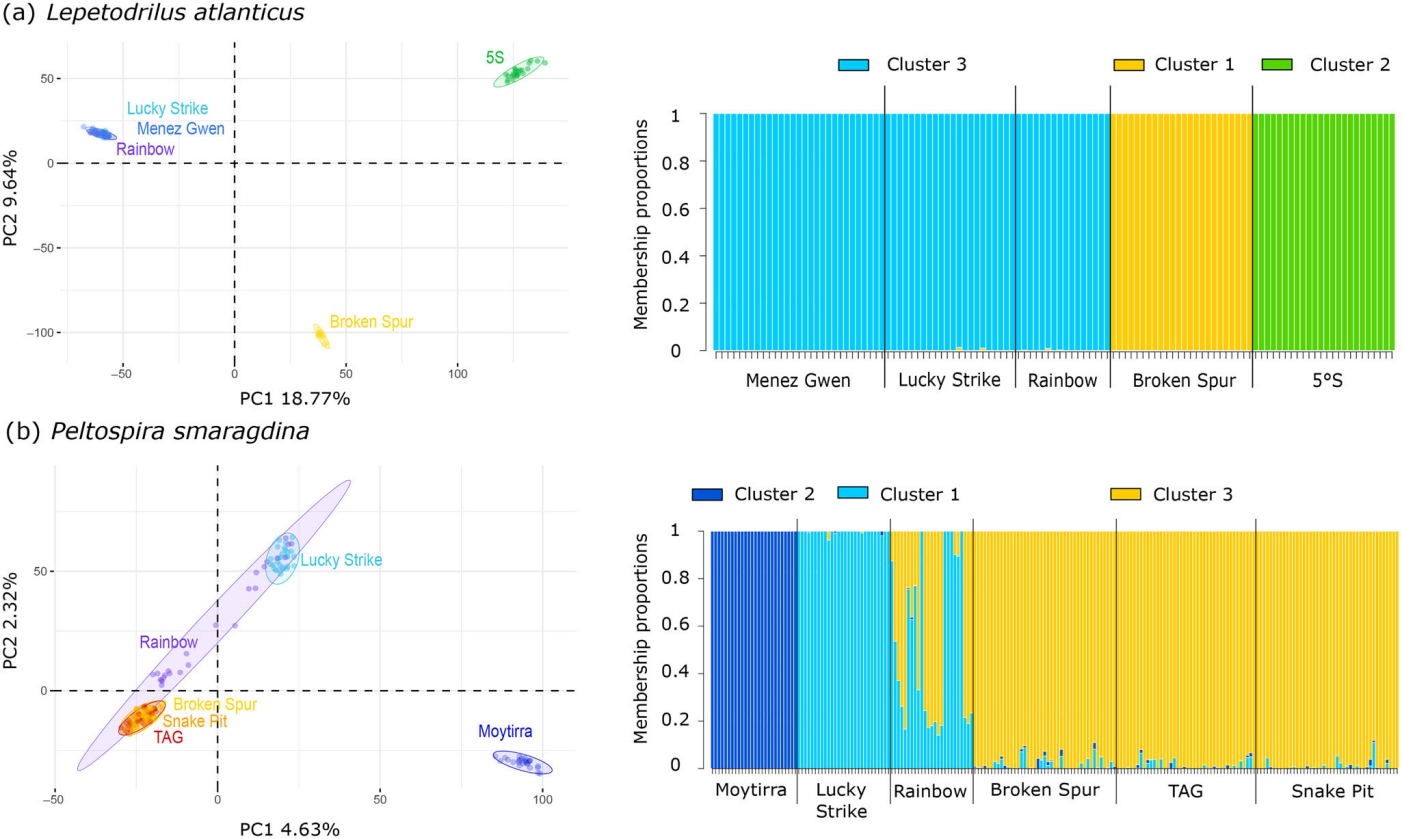
Results of principal component (left) and admixture (right) analyses performed on the single‐nucleotide polymorphisms (SNPs) datasets for (a) *Lepetodrilus atlanticus* (15529 SNPs) and (b) *Peltospira smaragdina* (16603 SNPs).

For *L. atlanticus*, the PCA separated three genetic units on the first two components (18.77% and 9.64% of genetic variation explained) (Figure 3a; Appendix S16). Individuals from 5°S MAR, Broken Spur (29°N MAR), and the Azorean populations (Lucky Strike, Menez Gwen, Rainbow) were well differentiated (Figure 3A). ADMIXTURE analyses confirmed these three genetic clusters with the same geographic breaks (Figure 3a; Appendix S17). Pairwise *F*_ST_ values were high and significant between the three genetic units (around 0.4, *p* = 0) (Table 2) but not between Lucky Strike, Rainbow, and Menez Gwen, which belong to the same genetic cluster (*p* between 0.32 and 0.90) (Table 2).

**TABLE 2:** For *Lepetodrilus atlanticus* and *Peltospira smaragdina* populations, *F*_ST_ values.

*Lepetodrilus atlanticus*				
Population	Menez Gwen (CI)	Lucky Strike (CI)	Rainbow (CI)	Broken Spur (CI)
Lucky Strike	0.0001 (−0.0005 to 0.0009	0		
Rainbow	−0.0005 (−0.0014 to 0.0003	0.0002 (−0.0007 to 0.0012)	0	
Broken Spur	0.3763 (0.3685 to 0.3837)^*^	0.3783 (0.3703 to 0.3862)^*^	0.3817 (0.3738 to 0.3898)^*^	0
5°S	0.3631 (0.3569 to 0.3694)^*^	0.3563 (0.3502 to 0.3623)^*^	0.3508 (0.3446 to 0.3570)^*^	0.2992 (0.2927 to 0.3051)^*^

*Peltospira smaragdina*					
	Moytirra	Lucky Strike	Rainbow	Broken Spur	TAG
Lucky Strike	0.1437 (0.1387 to 0.1483)^*^	0			
Rainbow	0.1560 (0.1503 to 0.1615)^*^	0.0239 (0.0221 to 0.0259)^*^	0		
Broken Spur	0.1885 (0.1816 to 0.1954)^*^	0.0807 (0.0757 to 0.0862)^*^	0.0289 (0.0271 to 0.0307)^*^	0	
TAG	0.1886 (0.1814 to 0.1957)^*^	0.0807 (0.0757 to 0.0861)^*^	0.0289 (0.0269 to 0.0307)^*^	0.0013 (0.0008 to 0.0018)^*^	0
Snake Pit	0.1916 (0.1845 to 0.1988)^*^	0.0826 (0.0774 to 0.0878)^*^	0.0308 (0.0289 to 0.0327)^*^	0.0013 (0.0009 to 0.0017)^*^	−0.0003 (−0.0007 to 0.00001)

* Significantly different from zero values (null *p* values).

For *P. smaragdina*, the first components of the PCA explained 4.63% and 2.32% of the genetic variance (Appendix S16). Three clusters were observed: Moytirra, Lucky Strike, and a third group comprising the Broken Spur, TAG, and Snake Pit populations (Figure 3b). Rainbow individuals were either intermediate between the two latter groups or closer to the Lucky Strike group (Figure 3b). ADMIXTURE analyses confirmed these results, with Rainbow individuals showing membership proportions from 54% to 100% in Cluster 1 (Lucky Strike cluster) and from 63 to 100% in Cluster 3 (Broken Spur, TAG, and Snake Pit cluster) (Figure 3b; Appendix S17). Interestingly, none of the Rainbow individuals were assigned with 100% membership to the Broken Spur, TAG, and Snake Pit genetic unit (Figure 3b). Hybrid index analysis suggested the presence of backcrossed or F2 individuals at Rainbow (details in Appendix S18). Broken Spur, TAG, and Snake Pit individuals had a maximum of 4–10% of their genome assigned to the Moytirra or Lucky Strike genetic units, respectively, and only one individual sampled at Lucky Strike appeared to be slightly introgressed (Figure 3b). With the less statistically supported value of *K* = 4 (Appendix S19), backcrossed Rainbow individuals formed a separate group genetically close to the parental cluster (Figure 3b) because of their high ancestry membership to Broken Spur, TAG, and Snake Pit. Pairwise *F*_ST_ values between Moytirra and all other populations were high (*F*_ST_ = 0.2, *p* = 0) (Table 2), whereas they were much lower (*F*_ST_ = 0.001–0.08) (Table 2) though significant (*p* = 0) between other pairs. The only nonsignificant *F*_ST_ was found between TAG and Snake Pit (Table 2; *p* = 0.97).

### Demographic history

**FIGURE 4:**
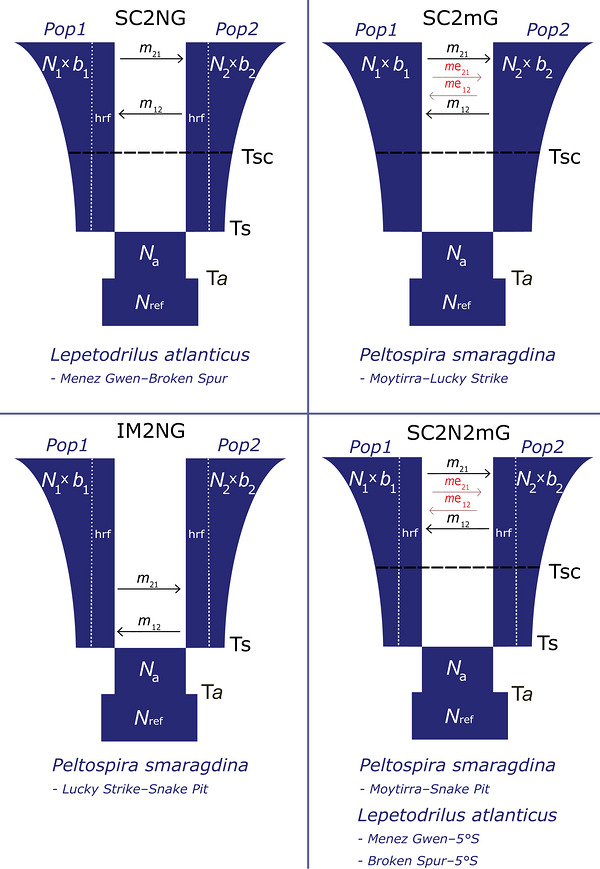
Representation of the most supported demographic models in dadi analyses for *Lepetodrilus atlanticus* and *Peltospira smaragdina*. Demographic parameters are detailed in Table 3.

For *L. atlanticus*, secondary contact models best fitted for the three pairs (SC2N2mG when 5°S MAR was considered, or SC2NG [Appendices S20 & S21]). All included population growth (G) and linked selection (2N) affecting 20–49% of loci (*Q*; Table 3; Figure 4). For pairs including 5°S MAR, heterogeneous gene flow along the genome (2 m) was supported, with a high proportion of barrier loci (1 − *P* = 97–99%) (Table 3). For these pairs, the time spent in strict isolation since the split of the ancestral population (*T*_s_) (Figure 4) was considerably higher than that of the Menez Gwen–Broken Spur pair (Table 3). Secondary contacts seemed to be more recent (*T*_sc_ < 900 generations) (Table 3). Assuming a generation time of 1 year, as hypothesized for other hydrothermal species (e.g., Tran Lu Y et al., 2022; Tyler & Young, 1999), secondary contacts occurred a few hundred years ago. Northward and southward restricted and unrestricted migration rates were low between all populations, although a southward migration seemed favored (restricted gene flow in all pairs, unrestricted gene flow between Menez Gwen and Broken Spur) (Table 3). The weak unrestricted gene flow occurred predominantly northward from 5°S to Broken Spur (see *p* values in Table 3). Population sizes estimated from pairs of populations cannot be interpreted as is because values of *N*_ref_ differed for the same population in different pairs. Nevertheless, they remained informative in terms of relative value, showing that 5°S MAR and Broken Spur had the largest and smallest population sizes, respectively (Table 3). The growth parameter (*b*) was greater than 1 for the Menez Gwen population, suggesting that this population was increasing in size, whereas the other two were likely decreasing (*b* < 1) (Table 3).

**TABLE 3:** Demographic parameters (SD) estimated using dadi software for *Lepetodrilus atlanticus* and *Peltospira smaragdina* population pairs (population 1–population 2).

	*Lepetodrilus atlanticus*			*Peltospira smaragdina*		
Population parameters	Menez Gwen–Broken Spur	Menez Gwen–5°S	Broken Spur–5°S	Moytirra–Lucky Strike	Moytirra–Snake Pit	Lucky Strike–Snake Pit
	SC2NG	SC2N2mG	SC2N2mG	SC2mG	SC2N2mG	IM2NG
nu_1_	1.430 (0.196)	1.054 (0.083)	0.917 (0.642)	2.611 (0.150)	1.779 (0.302)	0.301 (0.016)
nu_2_	0.936 (0.109)	4.678 (0.464)	2.173	8.478 (1.108)	8.944 (5.986)	1.214
nu_A_	7.303 (2.495)	0.454 (0.185)	9.992 (2.773)	0.394 (0.231)	1.043 (0.814)	8.98 (0.352)
*N*_ref_	3445	1262	599	1159	996	2422
*N*_1_	4926 (676.575)	1329 (105.004)	549 (384.706)	3027 (173.954)	1772 (300.433)	729 (38.128)
*N*_2_	3223 (376.556)	5902 (586.015)	1301	9826 (1284.693)	8908 (5961.684)	2940
*N*_a_	25155 (8595.353)	573 (233.615)	5982 (1661.456)	457 (267.889)	1038 (810.996)	21748 (853.328)
*T*_a_	8539 (2646.905)	1103 (318.231)	11900 (3445.962)	4143 (7954.332)	714 (616.079)	7957 (335.132)
*T*_s_	4255 (1937.841)	12709 (299.316)	10873 (2486.614)	10776 (617.013)	4083 (374.861)	794 (78.836)
*T*_sc_	1614 (279.293)	837 (59.409)	390 (13.222)	1182 (122.421)	586 (137.383)	NA
*b*_1_	1.644 (0.446)	1.272 (0.049)	0.393 (0.215)	0.356 (0.044)	0.351 (0.105)	7.443 (1.721)
*b*_2_	0.358 (0.115)	0.033 (0.008)	0.256	0.308 (0.091)	0.208 (0.364)	21.464 (6.157)
hrf	0.179 (0.017)	0.087 (0.014)	0.042 (0.001)	N.A.	0.124 (0.028)	0.044 (0.003)
*P*	N.A.	0.008 (0.051)	0.031 (0.135)	0.913 (0.018)	0.999 (0.136)	N.A.
*Q*	0.493 (0.131)	0.169 (0.021)	0.274 (0.029)	N.A.	0.255 (0.068)	0.233 (0.020)
m12 (north)	0.986 (0.146)	1.305 (0.150)	5.517 (0.489)	0.606 (0.082)	0.782 (1.222)	8.031 (0.695)
m21 (south)	2.314 (0.330)	1.954 (0.155)	1.018 (0.076)	1.935 (0.193)	4.084 (0.632)	1.113 (0.323)
me12 (north)	N.A.	0.138 (0.025)	0.323 (0.041)	0.068 (0.049)	0.726 (0.262)	NA
me21 (south)	N.A.	3.125 (0.211)	3.113 (0.114)	0.466 (0.066)	1.244 (0.524)	NA
M12 (north)	0.0001	0.0005	0.0046	0.0003	0.0004	0.0017
M21 (south)	0.0003	0.0008	0.0008	0.0008	0.0021	0.0002
Me12 (north)	NA	0.0001	0.0003	0.0000	0.0004	NA
Me21 (south)	NA	0.0012	0.0026	0.0002	0.0006	NA
Theta	2088.593 (180.520)	765.054 (30.447)	363.001 (48.819)	643.725 (38.352)	553.133	1345.138 (31.648)

*Note*: Compass directions correspond to gene flow direction along the ridge. Times (*T*) expressed in generations following calculations of *N*_ref_. Mutation rate is 10^−8^ per generation. Calculation details are in Appendix S10.

Abbreviations: *N*_1_, size of population 1 calculated as *N*_ref_ = *N*_1_ × nu_1_; *N*_2_, size of population 2 calculated as *N*_ref_ = *N*_2_ × nu_2_; *N*_ref_, size of the ancestral population after a demographic change; nu_1_, population size parameter estimate from dadi for population 1; nu_2_, population size parameter estimate from dadi for population 2; nu_A_, population size parameter estimate from dadi for the ancestral population; *T*_s_, time of strict divergence; *T*_a_, time before *T*_s_ at which a change in population size *N*_a_ occurred,; *T*_sc_, time of divergence with migration (*T*_s_ for IM models); *b_i_*, population *i*’s growth factor; hrf, Hill–Robertson factor (simulates linked selection); *P*, proportion of loci affected by unrestricted migration; *Q*, proportion of loci under linked selection; *m_ij_*, unrestricted migration rate from population *j* toward population *i*; me*_ij_*, restricted migration rate from population *j* toward population *i*; *M_ij_* and Me*_ij_*, migration rates corrected for population size (i.e., calculated as *m*/[2 × *N*_ref_] or me/[2 × *N*_ref_]) to represent the fraction of individuals in population *i* that are new migrants from population *j* in each generation.

For *P. smaragdina*, SC models were also supported for population pairs including Moytirra, whereas an isolation with migration (IM) model better fitted for the Lucky Strike–Snake Pit pair (Table 3; Figure 4; Appendices S20 & S22). Although an SC model was top‐ranked for Moytirra–Lucky Strike, AM and IM models also performed well (ΔAIC = 10) (Appendix S22). For the pairs including Moytirra, a high proportion (*P* = 91–99.9%) of loci were affected by unrestricted gene flow, whereas 26% of loci (*Q*) were under linked selection for the Moytirra–Snake Pit pair (Table 3). The IM2NG model for the Lucky Strike–Snake Pit pair included a similar proportion of loci under linked selection (*Q* = 23%) (Table 3). Splitting dates suggested that Moytirra became isolated several thousand generations ago (5000 < *T*_s_ < 10,000 generations) (Table 3). Secondary contacts were recent, particularly with the Snake Pit population (*T*_sc_ = 586 generations) (Table 3). Migration rates were low for all population pairs and mainly southward oriented when considering Moytirra but possibly northward oriented from Snake Pit to Lucky Strike (Table 3). When making relative size comparisons between populations, Moytirra has the smallest population size and Snake Pit the largest one (Table 3). Interestingly, the Moytirra population also exhibited the lowest heterozygosity (Appendix S15).

## DISCUSSION

### Genetic differentiation of vent populations along the MAR

Using microsatellites or mt*Cox1* gene, studies on hydrothermal shrimps *Rimicaris* spp. and limpets *Divia briandi* suggest strong gene flow along the whole MAR (Teixeira et al., 2012, 2013; Yahagi et al., 2019). In contrast, three genetic breakpoints were detected for *L. atlanticus* and *P. smaragdina* with mt*Cox1* and SNP markers. Although secondary contacts may have occurred between most population pairs, gene flow was strongly limited between Moytirra and Lucky Strike, Lucky Strike and Broken Spur, and Broken Spur and 5°S. Alvinocarid shrimp and *D. briandi* larvae are supposed to be planktotrophic and to vertically migrate in the water column, favoring extended larval life and long‐distance dispersal (Hernández‐Avila et al., 2015; Teixeira et al., 2012, 2013; Yahagi et al., 2017). *Peltospira smaragdina* and *L. atlanticus* may, instead, be characterized by lecithotrophy and larval development close to the seafloor (Chen et al., 2025; Tyler et al., 2008). This could favor dispersal through deeper, slower currents and explain the higher level of genetic differentiation observed in these species. However, similar genetic breaks with a hybrid zone were detected for bathymodioline mussels despite their expected high dispersal ability (Breusing et al., 2016; Mullineaux et al., 2005; O'Mullan et al., 2001; van der Heijden et al., 2012).

For the vent fauna, genetic breaks may, among other processes (e.g., natural selection), result from isolation by distance or the presence of physical barriers to dispersal (e.g., transform faults, microplates; Johnson et al., 2006; Matabos & Jollivet, 2019; Plouviez et al., 2009; Vrijenhoek, 2010). For *L. atlanticus*, around 5000 km separates the most genetically differentiated population (5°S, at both mt*Cox1* and SNPs) from all other populations. The offset of the Romanche fracture zone (2°N–2°S) and the equatorial cross‐Atlantic current at 1000 m depth may also contribute to the isolation of the northern and southern MAR (Yearsley et al. [2020] and references therein). Secondary contact models were supported between all pairs of populations with very low migration rates. For bathymodioline mussels, a contact zone between the southern and northern species occurs northward of these putative barriers to dispersal (Breusing et al., 2016, 2017). Although lepetodrilid gastropods exhibit oocyte sizes in the same range as bathymodioline mussels, whose larvae are planktotrophic (i.e., <90 µm) (Tyler & Young, 1999; Tyler et al., 2008), larval shell growth was limited during their time in the water column (up to 170 µm) (Mills et al., 2009). This suggests that larvae have limited vitelline reserves and do not feed for a long period of time in the plankton (Tyler et al., 2008). In addition, *L. atlanticus* has a rather low fecundity compared to other lepetodrilid species or bathymodioline mussels, which could contribute to a low larval exchange between vents (Laming et al., 2018; Tyler & Young, 1999; Tyler et al., 2008). Between the northern and southern MAR, the SC model estimated a long period of strict isolation, heterogeneous gene flow, and the presence of loci affected by linked selection, suggesting that potential genetic incompatibilities may have accumulated, preventing admixture. Interestingly, the separation of the Menez Gwen and Broken Spur populations seemed to be more recent than the separation of the 5°S population from the two others. This is consistent with the lower genetic differentiation and the lack of heterogeneous gene flow observed between Menez Gwen and Broken Spur than between the 5°S population and the others. We nevertheless cannot rule out that the choice of an SC model may be erroneous, due to incomplete lineage sorting caused by a very recent divergence, possibly enhanced by large population effective sizes (Gagnaire et al., 2015).

For *P. smaragdina*, the most differentiated population was Moytirra, which is less than 1000 km away from Lucky Strike. Deviation in oceanic circulation and changes in seafloor topography related to the presence of the Azores Triple Junction (Zhai et al., 2021) may hinder larval exchanges between these sites. The resulting shallow Azores plateau, extending over an area of more than 400 km, could constitute a depth‐related barrier for this species, given its absence from the Menez Gwen vent field, which is 800 m deep. Despite the apparent genetic isolation and low migration rates, secondary contacts between Moytirra and other populations were suggested. Reconnection may be too recent to allow inference of present‐day gene flow (*T*_sc_ < *T*_s_), or the long period of strict isolation of Moytirra may have led to genetic incompatibilities preventing any admixture (as suggested by the presence of heterogeneous gene flow). Nonnegligible proportions of loci were affected by linked selection, which is often associated with barrier loci (Stankowski et al., 2019). The choice of an SC model may also be biased by incomplete lineage sorting if populations have separated recently. Conversely, the IM model selected for the Snake Pit and Lucky Strike pair may be explained by the proximity of Lucky Strike to the contact zone (near Rainbow).

For both species, a genetic break occurred north of 29°N (Broken Spur), as observed for *Bathymodiolus* species for which dispersal distance is expected to be large due to long pelagic larval duration, larval planktotrophic development, and vertical migration (Breusing et al., 2016; Laming et al., 2018; Mullineaux et al., 2005; O'Mullan et al., 2001). The lack of active vents between the sampled sites may cause this genetic disruption if released larvae did not survive long enough to settle in favorable areas or were lost during their journey (Audzijonyte & Vrijenhoek, 2010). Modeling larval dispersal of *Bathymodiolus* species suggested that the observed genetic connectivity relied on undiscovered stepping stones because, otherwise, population connectivity was limited between sites more than 150 km apart (Breusing et al., 2016). The Oceanographer or Atlantis fracture zones offset the ridge between 29°N and 36°N and may impede gene flow. Particle dispersal simulations of highly dispersive larvae released at Lucky Strike Rainbow are, indeed, unlikely to reach Broken Spur, and such exchanges are even less likely when dispersal occurs near the seafloor due to slower currents or topographic barriers (Gary et al., 2020; McVeigh et al., 2017). The depth gradient from Menez Gwen (800 m) to Snake Pit (>3500 m) may also limit larvae settlement and post‐larvae survival (McClain & Etter, 2005; Sarrazin et al., 2022). *Peltospira smaragdina* is absent from Menez Gwen despite the presence of suitable habitats, whereas larvae originating from Lucky Strike should be able to reach Menez Gwen when migrating in the hydrothermal plume (Vic et al., 2018). Recent information on the larval biology of *P. smaragdina* and *P. gargantua* (Chen et al., 2025, F. Pradillon, personal observation) suggested that larval development occurs close to the seafloor. This agrees with their large oocyte size and is possibly indicative of lecithotrophic larval development (M. Matabos, personal observation).

Habitat fragmentation may also be more pronounced for *L. atlanticus* associated with mussels because mussels are rare between Snake Pit and Rainbow and absent from Moytirra. Conversely, *P. smaragdina* colonizes bare substratum covered by mineral deposits or bacterial mats that develop on vent flanges and chimney walls, which are more abundant along the MAR (Sarrazin et al., 2022). The species harbors a putative endosymbiotic relationship, as observed for other species of the same genus (Chen et al., 2025; Portail et al., 2018), and might also graze on bacterial mats (Sarrazin et al., 2022), which could increase its range of suitable habitats. The low habitat suitability between Lucky Strike and Broken Spur for *L. atlanticus* may thus explain the higher genetic differentiation observed between those two vent fields when compared to *P. smaragdina*. Divergence was overall higher for *L. atlanticus*, with no detected admixed individuals, whereas hybrids were detected for *P. smaragdina*. The higher abundance of suitable habitats (chimney walls) for *P. smaragdina* as compared to those for *L. atlanticus* (mussel beds) may enhance connectivity.

### A multispecies hybrid zone in an area of reduced migration

Although migration rates estimated from the SC models selected for *P. smaragdina* were low, gene flow seemed to be mostly southward oriented. This directionality is also illustrated by the presence of putative parental individuals from Lucky Strike (very high membership of 99.9–100% to Cluster 1) at Rainbow. Southward‐oriented gene flow was suggested by the presence of a few individuals in TAG, Snake Pit, or Broken Spur with a small portion of their genome assigned to the Moytirra or Lucky Strike genetic clusters. Almost no admixture was detected in the latter two populations. This directionality, which may result from southward‐flowing currents in this area, also characterized bathymodioline vent mussels (Breusing et al., 2017; Lahaye et al., 2019; Vic et al., 2018). Individuals from Rainbow, for which ancestry was mostly assigned to the Broken Spur, TAG, and Snake Pit genetic cluster, suggested some northward‐oriented flow that probably ends there (almost no admixture at Lucky Strike). Although individuals with 100% of their genome assigned to Cluster 2 may occur at Rainbow, the absence of a parental type suggests that the contact zone is located south of Rainbow. Admixed larvae may flow northward to settle at this site. Bidirectional currents flowing along the flank of the rift valley may ensure larval fluxes (Khripounoff et al., 2008; Lahaye et al., 2019; Sarrazin et al., 2020; Vic et al., 2018).

Broken Spur and Snake Pit represent hybrid zones between the vent mussels *Bathymodiolus azoricus* and *Bathymodiolus puteoserpentis*, but not Rainbow. Because no F1 hybrids were found at Broken Spur for these species, the hybrid zone was hypothesized to occur in an unknown location, south of Rainbow (Breusing et al., 2016, 2017; Faure et al., 2009; O'Mullan et al., 2001). The genetic structure of bathymodioline mussels and *P. smaragdina* suggests that the region located around Broken Spur represents a multispecies hybrid zone. Hybrid zones often coincide with barriers to dispersal, and the width of allelic clines depends on a balance between the parental homozygote migration rate and the strength of the selection against hybrids (Barton, 1979). They can also coincide with ecological barriers to dispersal (Bierne et al., 2011). The co‐occurrence of hybrid zones for several species at the same location supports the idea that this ridge portion represents an area of less migration. Multispecies hydrothermal hybrid zones were recently discovered in the western Pacific near the Woodlark Ridge, in a region where larval dispersal is minimal (Mitarai et al., 2016; Poitrimol et al., 2022; Tran Lu Y et al., 2025), and near the Easter Island microplate, which represents a barrier to dispersal between the EPR and the Antarctic–Pacific Ridge (Johnson et al., 2013). Hydrothermal hybrid zones thus seem common, which suggests that tectonic rearrangements play a role in evolutionary processes, promoting isolation in allopatry and secondary contacts.

### Conservation implications

The ISA is leading the establishment of regional environmental management plans with interspaced areas of no mining along the MAR (Dunn et al., 2018). So far, these plans have not accounted for species genetic variability and population fragmentation. Some vent fields lie within areas identified as vulnerable marine ecosystems and ecologically or biologically significant areas, but, to date, only the vent fields in Portuguese waters are legally protected (i.e., Lucky Strike, Rainbow, Menez, and Gwen) (Combes et al., 2021; Menini & Van Dover, 2019). Vent fields located outside national jurisdiction (Logatchev, Semienov, TAG, Snake Pit, Broken Spur) are within exploration contracts granted to the French, Polish, and Russian governments (ISA, 2023), although they are listed as “sites in need of protection” where no mining should occur (Blanchard & Gollner, 2022).

The pronounced genetic structure in vent gastropods suggests that hundreds of generations of genetic exchanges were not sufficient for the rehomogenization of allele frequencies after secondary contacts. Exchanges may thus be sporadic and rare, or adaptation to depth may represent a barrier. Populations located on either side of genetic breaks might be viewed as isolated or semi‐isolated, and poorly connected sites may not be able to recover from mining‐induced disturbances or local extinction. The presumably isolated populations of Moytirra for *P. smaragdina* and 5°S and Broken Spur for *L. atlanticus* may thus need specific conservation measures because they are likely to shelter unique gene pools.

Broken Spur was previously mentioned as an important area for conservation, due to the hybrid zone identified for *Bathymodiolus* species (Dunn et al., 2018). The discovery of a hybrid zone for *P. smaragdina* in the present study suggests that the area located between 26°N and 35°N MAR is a multispecies hybrid zone. Beyond the local protection of source populations (e.g., Lucky Strike) that ensure the replenishment of hybrid populations and of areas of admixture (e.g., Rainbow), we recommend maintaining an average distance of less than 100 km between favorable protected habitats. This should apply to the populations that seemed well connected (Broken Spur, TAG, and Snake Pit for *P. smaragdina*; Menez Gwen, Lucky Strike, and Rainbow for *L. atlanticus*). Although Azorean populations are part of the marine natural reserves, where deepwater fishing activities and resource exploitation are prohibited (Menini & Van Dover, 2019), other sites in the French exploratory mining permit area (e.g., TAG, Snake Pit) may need immediate protection.

Because vent species exhibit differential connectivity patterns, additional genetic studies at the genomic scale for other MAR vent species would be needed to account for the less dispersive species (e.g., polychaetes) (Breusing et al., 2016; Hilario et al., 2015; Poitrimol et al., 2022). Conservation planning should also consider the connectivity decrease that would be caused by mining activities through habitat loss and dispersal disruption. Combining genetic data and the modeling of larval dispersal, as performed for *Bathymodiolus* species (Breusing et al., 2016), is of great utility to disentangle the roles played by habitat availability and dispersal strategy. Finally, because connectivity can evolve over time (sources can become sink populations and vice versa, in an environment that becomes exploitable by humans), protection plans should ideally be designed based on information collected at multiple points in time, and biodiversity and connectivity should be monitored together (Balbar & Metaxas, 2019). This is particularly relevant in the context of global changes, as species ranges, ecosystem functioning, spawning phenology, survival, and other parameters may change, thereby affecting connectivity patterns.

## AUTHOR CONTRIBUTIONS

M.M., D.J., and F.P. designed the research. E.P., C.D.T., S.R., S.F., E.O., and D.J. performed genomic libraries and sequence data filtering. J.C. and P.C. collected and provided samples from Moytirra (*P. smaragdina*: 45°N/MAR). E.P. conducted population genetic and demographic inference analyses, with the support of A.T.L. and D.J. E.P. wrote the first version of the manuscript. All authors provided scientific advice and improved the manuscript.

## Supporting information




**Appendices S5–S11, S13, S14**, and **S16–S20**

**Appendices S1–S4, S12, S15, S21**, and **S22**

## Data Availability

The ddRAD sequences generated in this study are available from the European Nucleotide Archive database (projects PRJEB61173 and PRJEB50821). mt*Cox1* sequences are available from the European Nucleotide Archive database with accessions OZ181773‐OZ181845 (EMBL‐EBI Study PRJEB50821) for *P. smaragdina* and accessions OZ181846‐OZ181879 (EMBL‐EBI Study PRJEB61173) for *L. atlanticus* (Appendix S3). Sample information is in Appendix S3. A. Tran Lu Y scripts for dadi execution are available from https://github.com/Atranluy/Scripts‐Ifremeria/tree/Main/Dadi_scripts. The modified dadi 2.1 version used is available from https://gitlab.mbb.univ‐montp2.fr/khalid/dadi/‐/tree/master. The VCF files are available from the PANGAEA database (Jollivet et al., 2023).
