# Supplementary material for: Conservation implications of low contemporary connectivity along the Mid‐Atlantic Ridge in hydrothermal vent gastropods
Source: Conserv Biol. 2026 Apr 24;40(4):e70284. doi: 10.1111/cobi.70284 (PMC13392762; doi:10.1111/cobi.70284)
Supplement: Supplementary file 1 — Appendices S5–S11, S13, S14, and S16–S20 [file COBI-40-e70284-s001.docx]

**Conservation implications of low contemporary connectivity along the Mid Atlantic Ridge in hydrothermal vent gastropods: assessment from genomic data.**

[**Details of mtCox1 PCRs** 4](#_Toc204864119)

[**Details of Stacks parameters optimisation and SNP filtering** 4](#_Toc204864120)

[Figure S1: Stacks parameters optimization for *Peltospira smaragdina*. A) Evolution of the total number of assembled de novo loci and of the number of polymorphic loci among those according to *M* and *n* parameters values. B) Evolution of the total number of SNPs (several per loci) according to *M* and *n* parameters values. C) Number of gained (or lost if negative) polymorphic loci between two *M* and *n* parameters values. D) Percentage of loci showing specific number of SNPs according to *M* and *n* parameters values. 5](#_Toc204864121)

[Figure S2: Stacks parameters optimization for *Lepetodrilus atlanticus*. A) Evolution of the total number of assembled de novo loci and of the number of polymorphic loci among those according to *M* and *n* parameters values. B) Evolution of the total number of SNPs (several per loci) according to *M* and *n* parameters values. C) Number of gained (or lost if negative) polymorphic loci between two *M* and *n* parameters values. D) Percentage of loci showing specific number of SNPs according to *M* and *n* parameters values. 6](#_Toc204864122)

[Figure S3: Pcadapt scree-plot and projections used to choose the number of PCs to keep in outlier detection analysis performed for *L. atlanticus*. The proportion of explained variance reaching zero from 4 PCs, we kept 4 PCs to be conservative and carefully exclude all loci that may be outliers. 7](#_Toc204864123)

[Figure S4: Pcadapt scree-plot and projections used to choose the number of PCs to keep in outlier detection analysis performed for *P. smaragdina*. The proportion of explained variance reaching zero from 4 PCs, we kept 4 PCs to be conservative and carefully exclude all loci that may be outliers. 8](#_Toc204864124)

[**Details of demographic models** 9](#_Toc204864125)

[**Details of demographic parameters calculation** 9](#_Toc204864126)

[Figure S5: ASAP results using the Kimura 80 substitution model for *L. atlanticus*. Right: histogram of distances. Left: Ranked partitions and their associated metrics: asap-score (mean of p-value and W ranks), p-values of partitions and associated ranks, width of the barcode gap W and associated ranks, threshold distance (see Puillandre et al. 2021 for details). 11](#_Toc204864127)

[Figure S6: ASAP results using the Kimura 80 substitution model for *P. smaragdina*. Right: histogram of distances. Left: Ranked partitions and their associated metrics: asap-score (mean of p-value and W ranks), p-values of partitions and associated ranks, width of the barcode gap W and associated ranks, threshold distance (see Puillandre et al. 2021 for details). 11](#_Toc204864128)

[Figure S7: A) Variance explained by each PC of the PCA performed on non-outlier loci for *L. atlanticus*. B) Variance explained by each PC of the PCA performed on non-outlier loci for *P. smaragdina*. 12](#_Toc204864129)

[Figure S8: Average CV-errors values across the 10 runs of ADMIXTURE for *Peltospira smaragdina* (left) and *Lepetodrilus atlanticus* (right), sampled along the Mid-Atlantic Ridge. 13](#_Toc204864130)

[**Details of hybridization analyses for *Peltospira smaragdina*.** 14](#_Toc204864131)

[Figure S9: Triangle plot of interspecific heterozygosity versus hybrid index for *Peltospira smaragdina* sampled along the Mid-Atlantic Ridge. 14](#_Toc204864132)

[Figure S10: Membership proportions of each individual in each genetic cluster obtained from ADMIXTURE when considering K=4 for *Peltospira smaragdina.* 15](#_Toc204864133)

[Figure S11: Join allele frequency spectrums obtained between all pairs of populations for each species. A) *Lepetodrilus atlanticus*, B) *Peltospira smaragdina*. 16](#_Toc204864134)

[Figure S12: Residuals of the fit of the best simulated model on the data for all pairs of populations for each species. A) *Lepetodrilus atlanticus*, B) *Peltospira smaragdin* 17](#_Toc204864135)

**Appendix S5 : Details of mtCox1 PCRs**

PCRs were performed in a final volume of 50 µL with 40 ng of DNA, 2X GoTaq® reaction buffer (Promega), 2 mM MgCl2, 0.1 mM of each dNTP, 0.3 mM of each forward and reverse Folmer primers and 1 U of GoTaq ® polymerase. The thermal profile consisted of 3 min of initial denaturation (95°C), followed by 35 or 40 cycles (for *P. smaragdina* and *L. atlanticus*, respectively) of denaturation (1 min, 95°C), annealing (35 s at 50°C) and extension (2 min, 72°C), with a final extension 10 min at 72°C. PCR products were checked on 1.5% agarose gels with ethidium bromide. In each plate, one negative and one positive controls were included.

Appendix S6 : **Details of Stacks parameters optimisation and SNP filtering**

A de novo assembly was then performed following the r0.80 procedure detailed in Paris et al. (2017) and Rochette and Catchen (2017) to optimize Stacks parameters. The *denovo_map.pl* function was thus ran on a subset of 30 and 60 samples representing the samples with the highest yield of reads from all vent localities for *L. atlanticus* and *P. smaragdina*, respectively, to generate a loci catalogue. Values from 1 to 9 were tested for the number of mismatches allowed between reads to create a stack within individuals (*-M*) and the number of mismatches allowed between individuals to group stacks (*-n*) with *M* = *n*. The minimum number of reads required to generate an initial stack (*-m*) was fixed to 3. The minimum percentage of individuals that share a RAD locus for a given population in the *populations* module was set to *-r* = 0.80. The optimal values for *-M* = *-n* were identified as the ones from which the number of polymorphic loci, the number of SNPs and the percentage of loci showing specific number of SNPs started to plateau. The selected values were -M and -n = 5 and 3 for *L. atlanticus* and for *P. smaragdina*, respectively (see Supplementary Figures S1 and S2).

The dataset obtained after the *de novo* assembly (see Main text) was then filtered using VCFtools (v.0.1.16) (Danecek et al. 2011) and R v.4.1.0 (R Core Team 2021). To eliminate potential unfiltered paralogs SNPs with a maximum read coverage greater than twice the average read coverage across nucleotide sites (i.e. as high as 50 and 48 for *L. atlanticus* and *P. smaragdina*, respectively) were excluded. A minimum coverage of 10 reads was also required. Using triplicates, we identified and excluded SNPs that were erroneously genotyped along the genome (i.e. if the same SNP was genotyped differently between replicated individuals, it was excluded from the dataset for all individuals). The replicate showing the lowest percentage of missing data was then kept in the dataset. SNPs and individuals with more than 10% of missing data were also excluded.

After keeping only one SNP per locus, outlier SNPs were excluded using the R package pcadapt v. 4.3.3 (Luu et al. 2017, Privé et al. 2020). For each species, four principal components (PCs) were used to eliminate outliers (see Supplementary Figures S3 and S4) following the Cattell’s rule (i.e. the optimal number of PCs is given from the break line in the scree-plot, with the following PCs accounting only for random variation). All SNPs showing p-values < 0.05, after adjustment using q-values, a false discovery rate (Benjamini and Hochberg 1995) or Bonferroni correction, were excluded from further analyses.


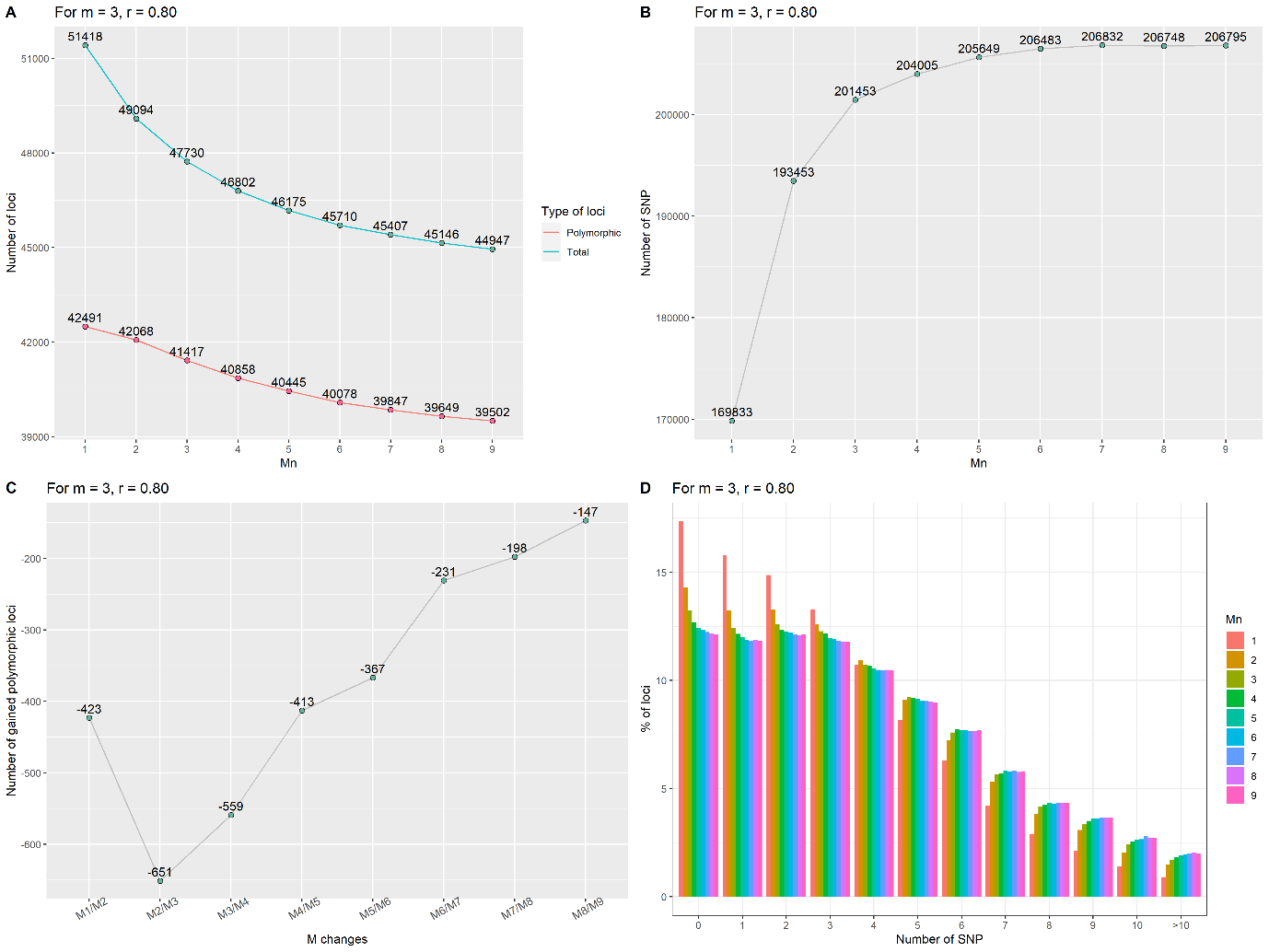


Figure 1: Stacks parameters optimization for *Peltospira smaragdina*. A) Evolution of the total number of assembled de novo loci and of the number of polymorphic loci among those according to *M* and *n* parameters values. B) Evolution of the total number of SNPs (several per loci) according to *M* and *n* parameters values. C) Number of gained (or lost if negative) polymorphic loci between two *M* and *n* parameters values. D) Percentage of loci showing specific number of SNPs according to *M* and *n* parameters values.


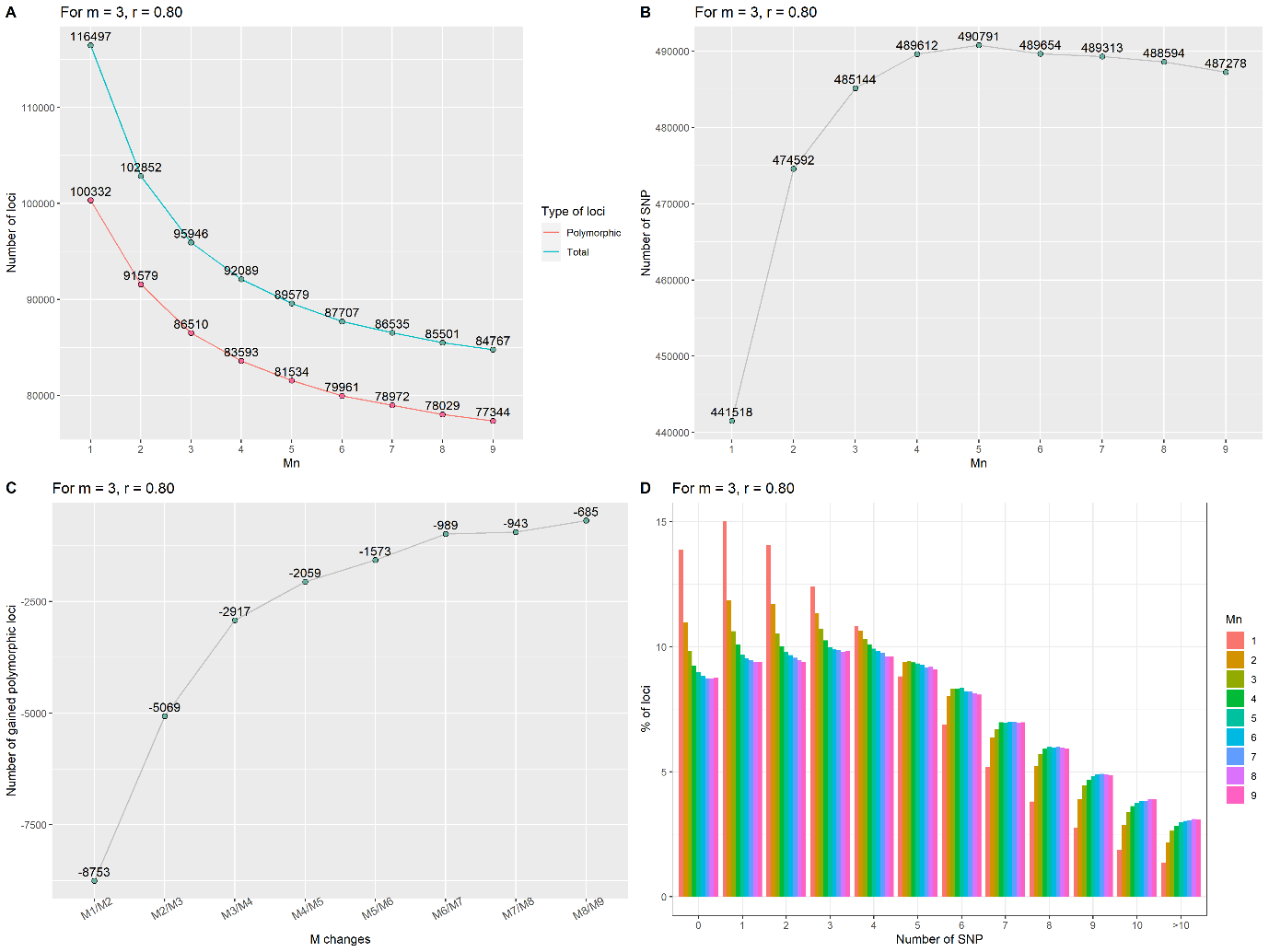


Figure 2: Stacks parameters optimization for *Lepetodrilus atlanticus*. A) Evolution of the total number of assembled de novo loci and of the number of polymorphic loci among those according to *M* and *n* parameters values. B) Evolution of the total number of SNPs (several per loci) according to *M* and *n* parameters values. C) Number of gained (or lost if negative) polymorphic loci between two *M* and *n* parameters values. D) Percentage of loci showing specific number of SNPs according to *M* and *n* parameters values.


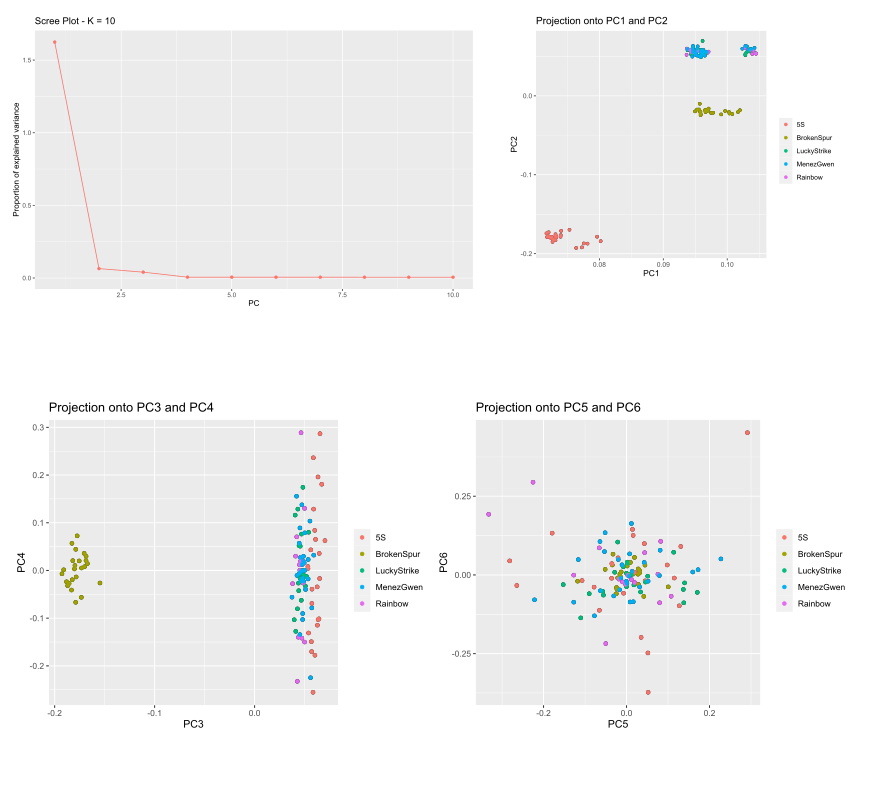


Appendix S7: Pcadapt scree-plot and projections used to choose the number of PCs to keep in outlier detection analysis performed for *L. atlanticus*. The proportion of explained variance reaching zero from 4 PCs, we kept 4 PCs to be conservative and carefully exclude all loci that may be outliers.


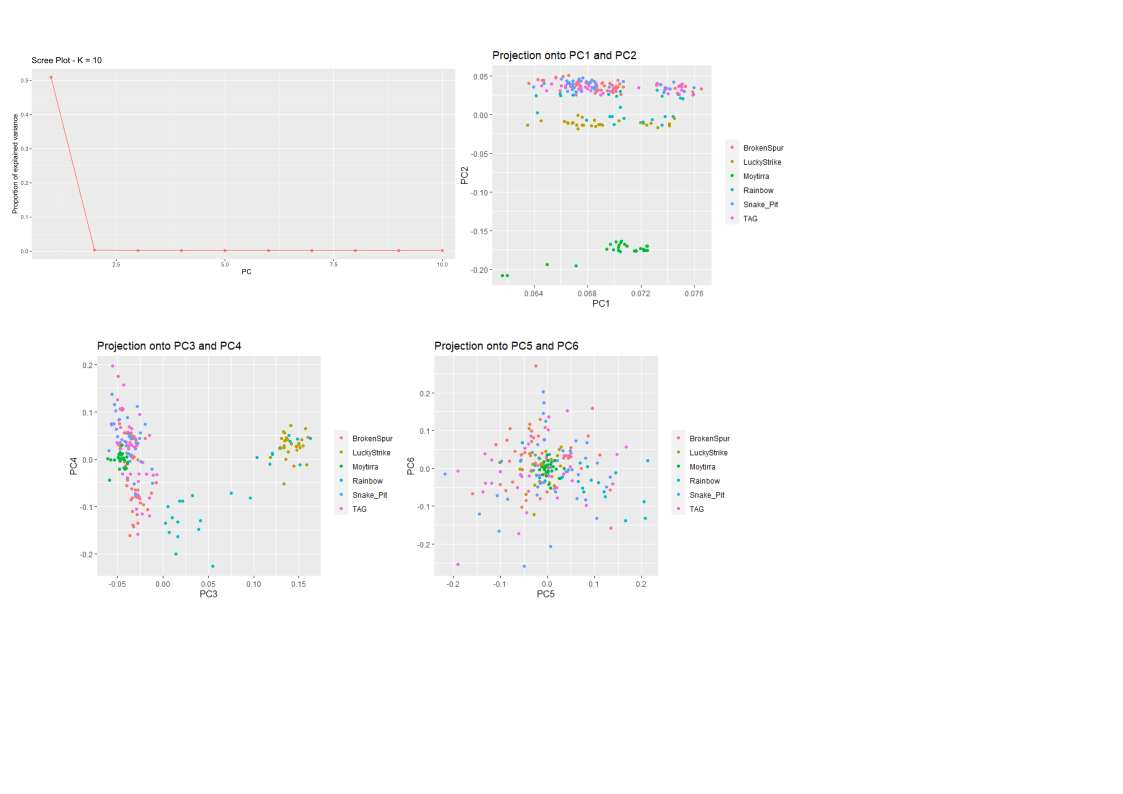


Appendix S8: Pcadapt scree-plot and projections used to choose the number of PCs to keep in outlier detection analysis performed for *P. smaragdina*. The proportion of explained variance reaching zero from 4 PCs, we kept 4 PCs to be conservative and carefully exclude all loci that may be outliers.

Appendix S9 : **Details of demographic models**

Following Rougeux et al. (2017) and Tran Lu Y et al. (2022), we considered 28 demographic scenarios that derived from four basic models: strict isolation (SI), isolation with migration (IM), ancient migration (AM), and secondary contact (SC) using a folded (no outgroup) JAFS (see <https://github.com/Atranluy/Scripts-Ifremeria/tree/Main/Dadi_scripts> for scripts by A. Tran Lu Y). Each model represented a situation in which an ancestral population of size N_anc_ splits in two sister populations of effective size N_1_ and N_2_ following a demographic change. Depending on the population model chosen, the time spent since the initial split of the ancestral populations into two daughter populations with (Tsm: IM) or without migration (Ts: SI) can be divided into two components: a period of past migration (Tam: AM) before the complete isolation (Ts) of the two daughter populations or a period of secondary contact migration (Tsc: SC) after a period (Ts) of strict isolation. In models where migration could occur (IM, AM and SC), directional migration was allowed at rates M_12_ and M_21_ from population 2 to population 1 and vice versa. Other modelled processes include independent demographic changes in the ancestral population size and in the two daughter populations after the initial split (growth parameters G), the effect of linked selection over a certain fraction of the genome (2N) and the effect of semipermeable genetic barriers (i.e., partial reproductive isolation, barrier loci) over a certain fraction of the genome (2m). As in Tran Lu Y et al. (2022), G was only allowed to vary during the migration phase (Tsm, Tam or Tsc) to dissociate the effects of the effective population size (drift) and migration (gene flow) (see: Supplementary Figure S7 in Tran Lu Y et al. (2022) for a graphical representation of some scenarios).

Appendix S10 : **Details of demographic parameters calculation**

According to Lynch (2010) and Tran Lu Y et al. (2022), we chose a mutation rate per site per generation µ equal to 10^-8^. In *dadi*, time and population size parameters are given in units of 2*N_ref_ generations and N_ref_, respectively. N_ref_ represent the size of the ancestral population after a demographic change and is estimated by ­­­θ / (4*µ*L) where θ is estimated by *dadi* and L represents the total length of the DNA sequence used in the analysis. In SNPs datasets, L = (*z***y***RADsize*)/*x* where *z* represents the number of SNPs used, *y* the number of RAD-tags (loci), *RADsize* the mean length of RAD-tags and *x* the initial number of SNPs. Thus, isolation times with or without migration in years were multiplied by 2*N_ref_ assuming a generation time of one year and population sizes (N_i_) were converted in numbers of individuals by multiplying N_u_i by N_ref_. Migration rates are expressed in units of m_ij_ = 2*N_ref_*M_ij_ where M_ij_ is the fraction of individuals in population i that are new migrants from population j in each generation. M_ij_ was thus calculated as m_ij_/(2*N_ref_). Restricted migration rates in 2m models are expressed in the same units and thus calculated the same way. For *Lepetodrilus atlanticus*, to estimate both population sizes and divergence times, the average RADsize was 275.92 bp, the number of initial (x) and retained (one SNP per RADTag, z=y) SNPs were 43 895 and 15 529, respectively, resulting in L= 1 515 846. For *Peltospira smaragdina*, the population sizes and the divergence times were estimated with an average RADsize of 264.05 bp, a number of initial (x) and retained (one SNP par RADTag, z=y) SNPs of 52 423 and 16 603, resulting in L= 1 388 473.


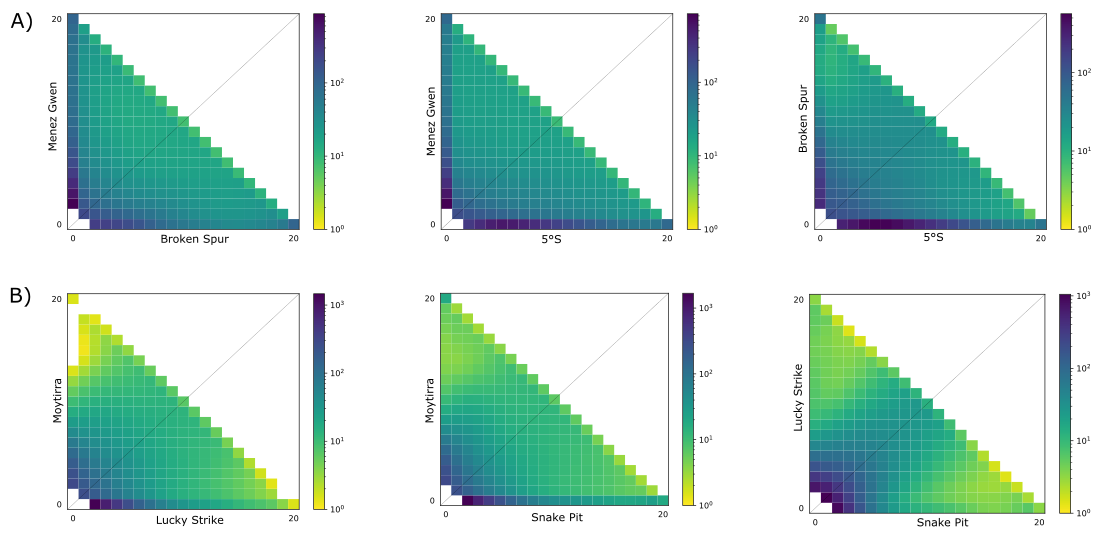


Appendix S11: Join allele frequency spectrums obtained between all pairs of populations for each species. A) *Lepetodrilus atlanticus*, B) *Peltospira smaragdina*.


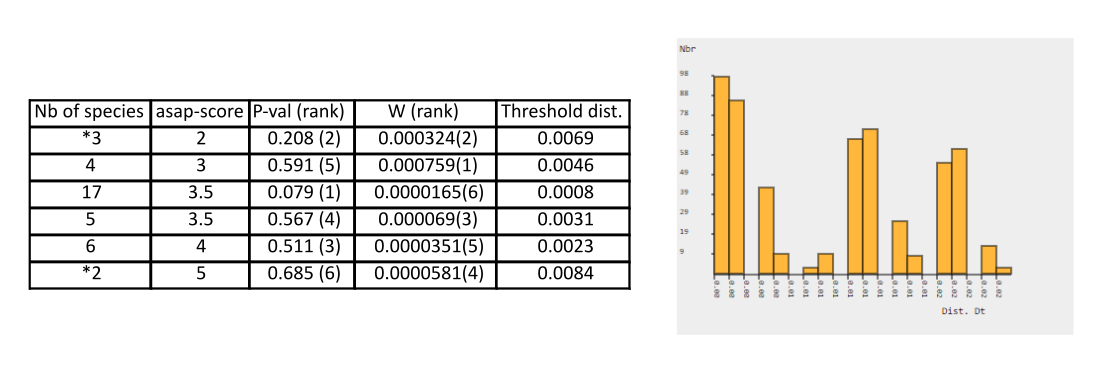


Appendix S13: ASAP results using the Kimura 80 substitution model for *L. atlanticus*. Right: histogram of distances. Left: Ranked partitions and their associated metrics: asap-score (mean of p-value and W ranks), p-values of partitions and associated ranks, width of the barcode gap W and associated ranks, threshold distance (see Puillandre et al. 2021 for details).


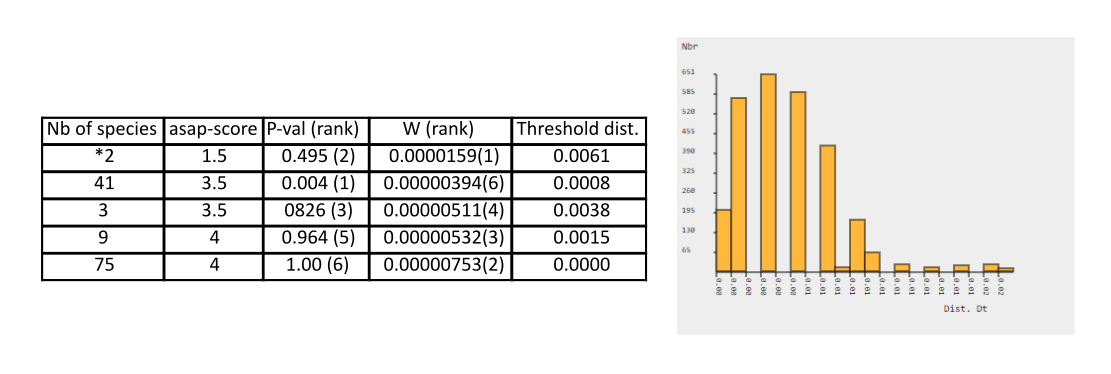


Appendix S14: ASAP results using the Kimura 80 substitution model for *P. smaragdina*. Right: histogram of distances. Left: Ranked partitions and their associated metrics: asap-score (mean of p-value and W ranks), p-values of partitions and associated ranks, width of the barcode gap W and associated ranks, threshold distance (see Puillandre et al. 2021 for details).

| A)  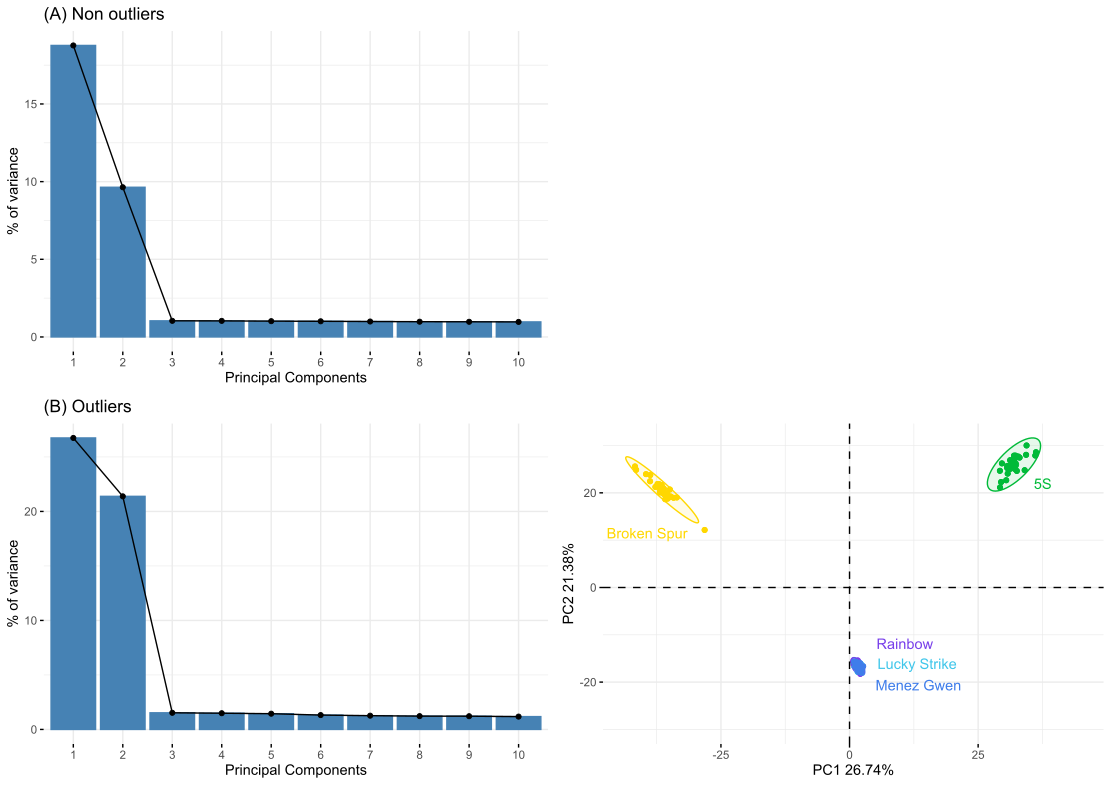 | B)  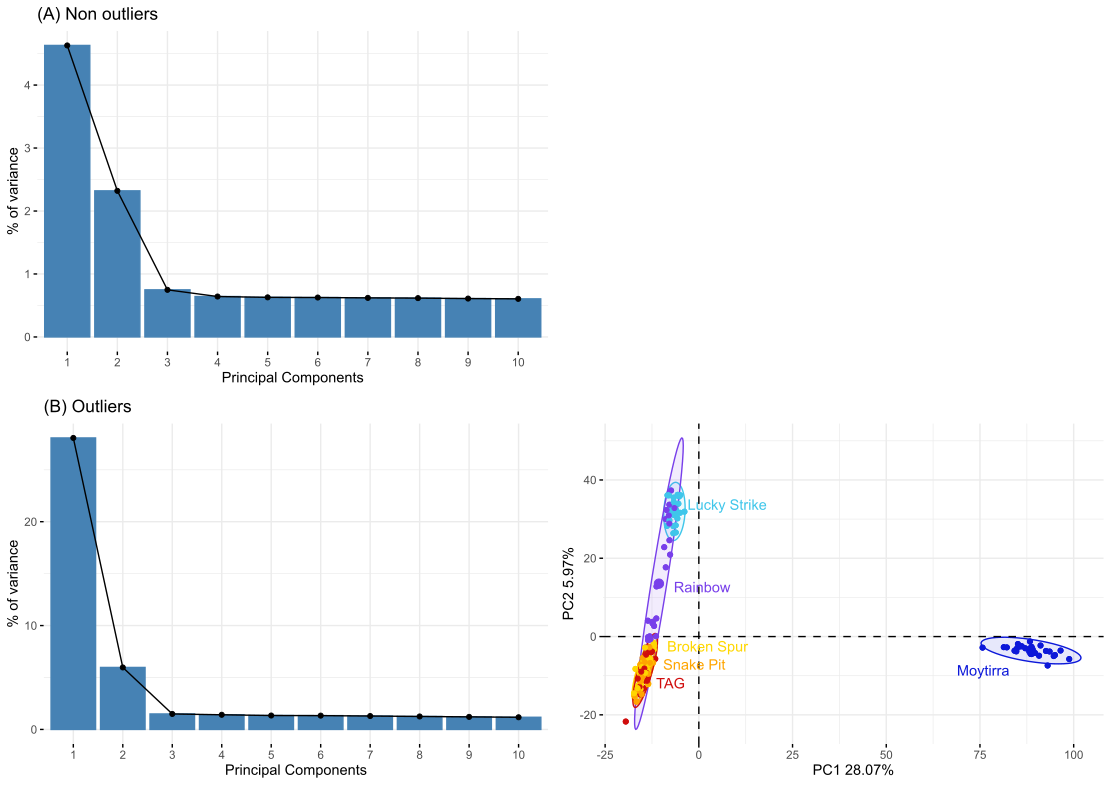 |
| --- | --- |

Appendix S16: A) Variance explained by each PC of the PCA performed on non-outlier loci for *L. atlanticus*. B) Variance explained by each PC of the PCA performed on non-outlier loci for *P. smaragdina*.


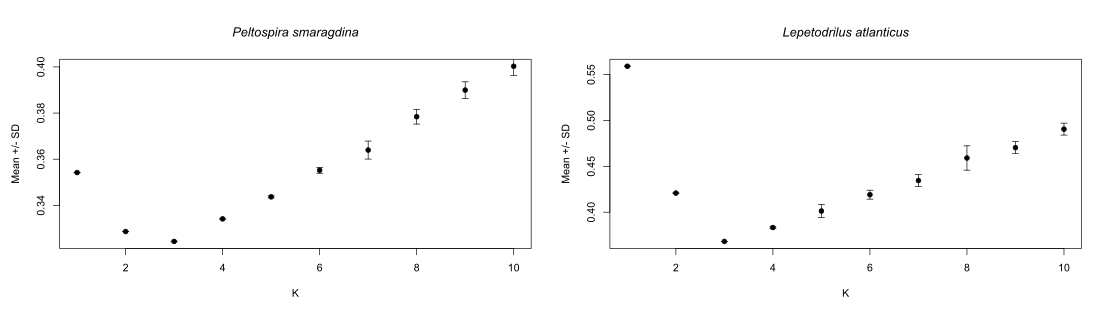


Appendix S17: Average CV-errors values across the 10 runs of ADMIXTURE for *Peltospira smaragdina* (left) and *Lepetodrilus atlanticus* (right), sampled along the Mid-Atlantic Ridge.

Appendix **S18 : Details of hybridization analyses for *Peltospira smaragdina*.**

In order to determine if hybrids were present between the genetic clusters formed by Lucky Strike and TAG/Snake Pit/Broken Spur populations, we relied on hybrid index, interclass heterozygosity and triangle plots. Using the R package *triangulaR* (Wiens & Colella 2024^^[[1]](#footnote-1)^^) we kept only SNPs showing allele frequency difference higher than 0.8 (n=54 SNP). Individuals from Moytirra were excluded from this analysis since admixture between this population and the other ones was lacking or seemed ancient or rare. Hybrid index and interclass heterozygosity values suggested that Lucky Strike and Broken/TAG/Snake Pit populations indeed formed parental populations while several individuals from Rainbow had position resembling backcrossed or F2 individuals (Supplementary Figure S9).


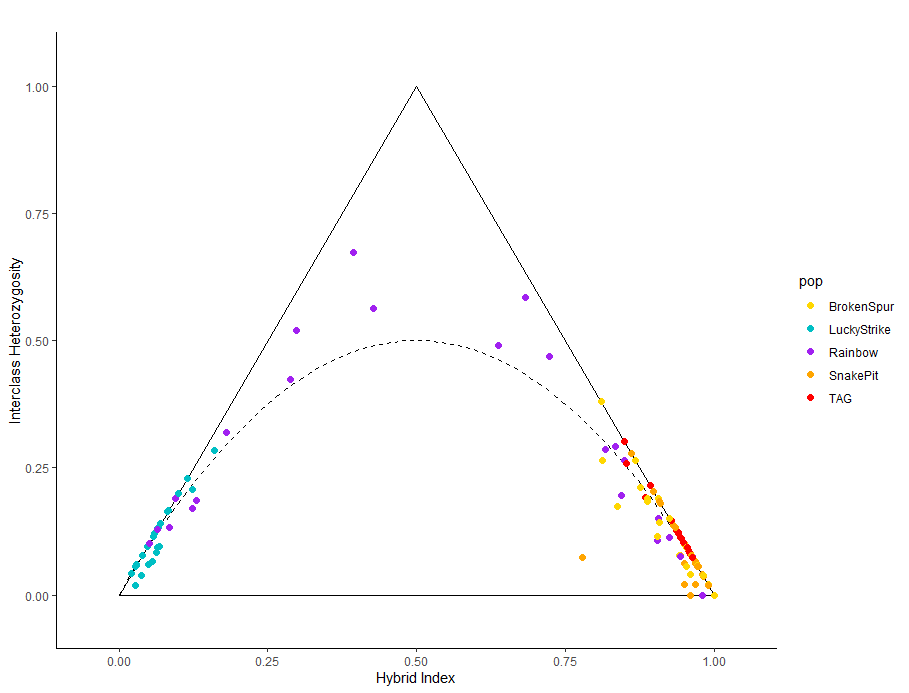


Figure 1: Triangle plot of interspecific heterozygosity versus hybrid index for *Peltospira smaragdina* sampled along the Mid-Atlantic Ridge.


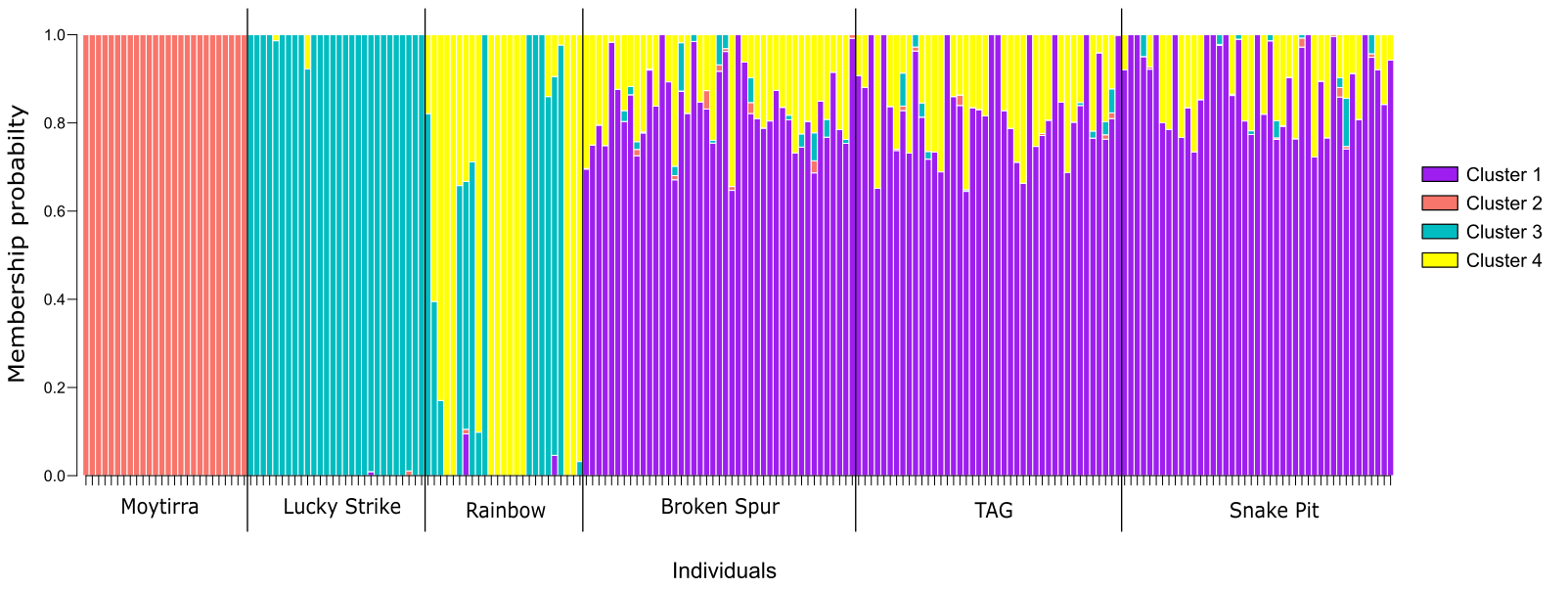


Appendix S19: Membership proportions of each individual in each genetic cluster obtained from ADMIXTURE when considering K=4 for *Peltospira smaragdina.*


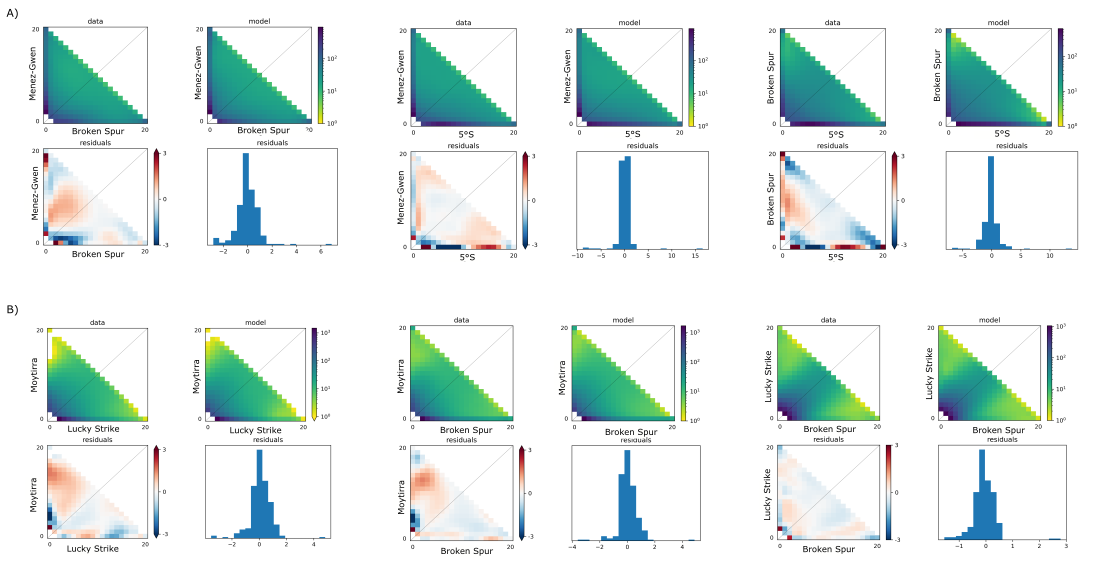


Appendix S20: Residuals of the fit of the best simulated model on the data for all pairs of populations for each species. A) *Lepetodrilus atlanticus*, B) *Peltospira smaragdin*

1. Wiens, B.J., Colella, J.P. (2024) triangulaR: an R package for identifying AIMs and building triangle plots using SNP data from hybrid zones. bioRxiv 2024.03.28.587167; doi: https://doi.org/10.1101/2024.03.28.587167 [↑](#footnote-ref-1)
